# Experiences of antenatal care practices to reduce stillbirth: surveys of women and healthcare professionals pre-post implementation of the Safer Baby Bundle

**DOI:** 10.1186/s12884-024-06712-8

**Published:** 2024-08-01

**Authors:** Christine Andrews, Frances M. Boyle, Ashley Pade, Philippa Middleton, David Ellwood, Adrienne Gordon, Miranda Davies-Tuck, Caroline Homer, Alison Griffin, Michael Nicholl, Kirstine Sketcher-Baker, Vicki Flenady

**Affiliations:** 1 Centre of Research Excellence in Stillbirth, Mater Research Institute, The University of Queensland, Brisbane, Australia; 2 Institute for Social Science Research, The University of Queensland, Brisbane, Australia; 3 South Australian Health and Medical Research Institute, Adelaide, Australia; 4 School of Medicine and Dentistry, Griffith University, Queensland, Australia; 5 School of Medicine, The University of Sydney, Sydney, Australia; 6 The Ritchie Centre, Hudson Institute of Medical Research, Melbourne, Australia; 7 Department of Obstetrics and Gynaecology, Monash University, Melbourne, Australia; 8 Burnet Institute, Melbourne, Australia; 9 QIMR Berghofer Medical Research Institute, Brisbane, Australia; 10 Clinical Excellence Commission, NSW Health, Sydney, Australia; 11 Clinical Excellence Queensland, Brisbane, Australia; 12 The University of Adelaide, Adelaide, Australia; 13 Gold Coast University Hospital, Gold Coast, QLD Australia

**Keywords:** Stillbirth, Care bundle, Survey, Antenatal care, Stillbirth prevention

## Abstract

**Background:**

The Safer Baby Bundle (SBB) aimed to reduce stillbirth rates in Australia through improving pregnancy care across five elements; smoking cessation, fetal growth restriction (FGR), decreased fetal movements (DFM), side sleeping in late pregnancy and decision making around timing of birth. We assessed experiences of women and healthcare professionals (HCPs) with antenatal care practices around the five elements.

**Methods:**

A pre-post study design using online surveys was employed to assess change in HCPs awareness, knowledge, and frequency of performing recommended practices (22 in total) and women’s experiences of care received related to reducing their chance of stillbirth. Women who had received antenatal care and HCPs (midwives and doctors) at services participating in the SBB implementation program in two Australian states were invited to participate. Surveys were distributed over January to July 2020 (pre) and August to December 2022 (post). Comparison of pre-post responses was undertaken using Fisher’s exact, Pearson’s chi-squared or Wilcoxon rank-sum tests.

**Results:**

1,225 women (pre-1096/post-129) and 1,415 HCPs (pre-1148/post-267, ≥ 83% midwives) completed the surveys. The frequency of HCPs performing best practice ‘all the time’ significantly improved post-SBB implementation across all elements including providing advice to women on side sleeping (20.4–79.4%, *p* < 0.001) and benefits of smoking cessation (54.5–74.5%, *p* < 0.001), provision of DFM brochure (43.2–85.1%, *p* < 0.001), risk assessments for FGR (59.2–84.1%, *p* < 0.001) and stillbirth (44.5–73.2%, *p* < 0.001). Practices around smoking cessation in general showed less improvement e.g. using the ‘Ask, Advise and Help’ brief advice model at each visit (15.6–20.3%, *p* = 0.088). Post-implementation more women recalled conversations about stillbirth and risk reduction (32.2–50.4%, *p* < 0.001) and most HCPs reported including these conversations in their routine care (35.1–83.0%, *p* < 0.001). Most HCPs agreed that the SBB had become part of their routine practice (85.0%).

**Conclusions:**

Implementation of the SBB was associated with improvements in practice across all targeted elements of care in stillbirth prevention including conversations with women around stillbirth risk reduction. Further consideration is needed around strategies to increase uptake of practices that were more resistant to change such as smoking cessation support.

**Trial registration:**

The Safer Baby Bundle Study was retrospectively registered on the Australian New Zealand Clinical Trials Registry database, ACTRN12619001777189, date assigned 16/12/2019.

**Supplementary Information:**

The online version contains supplementary material available at 10.1186/s12884-024-06712-8.

## Background

**Fig. 1:**
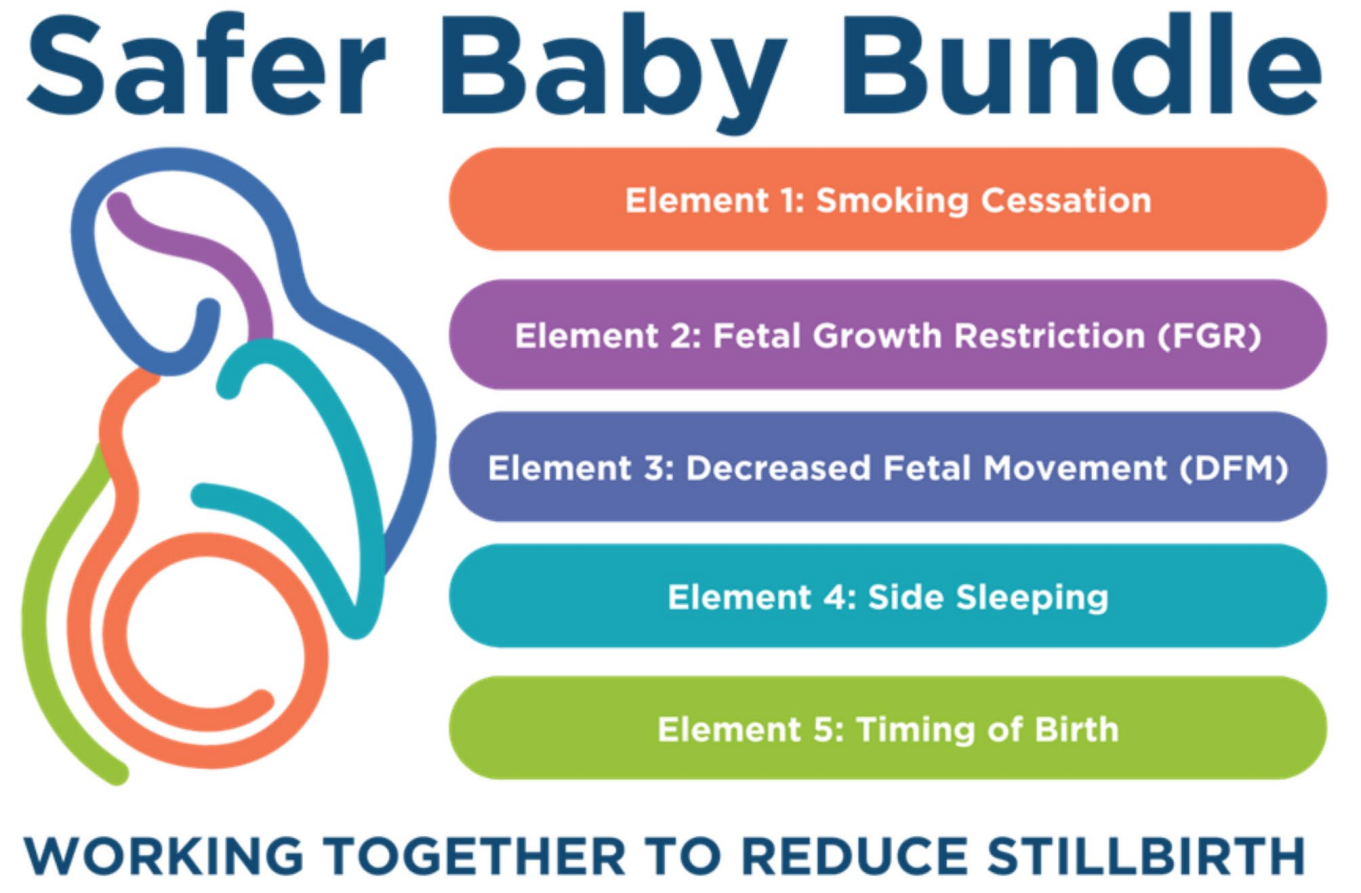
The five elements of the Australian Safer Baby Bundle

Variations in antenatal care can contribute to late gestational stillbirth which may have been avoidable. In an effort to reduce stillbirth across Australia the Safer Baby Bundle (SBB) has been implemented as a key national program for improving the quality of antenatal care [1–3]. SBB evidence-based recommendations for Australia and New Zealand [4–9] can address known gaps in care [10, 11]. The five SBB elements attend to recognised evidence practice gaps as shown in Fig. 1. Detailed best-practice recommendations for the five elements are described in the SBB Handbook and Resource Guide [12] and supported by the SBB eLearning [13]. This program of work is endorsed by professional organisations, parent advocacy networks, and Departments of Health partners for each state and territory, and was made freely available from October 2019.

Prior to the development of the SBB, substantial unnecessary variation in practice between maternity services was observed for the five care practices, including suboptimal uptake of guidelines [11]. The best practice recommendations which maternity services indicated they were least likely to perform ‘all the time’ were for smoking cessation support (Element 1, < 50%), fetal growth restriction (FGR) risk assessment (Element 2, < 40%) and providing guidance on safe going-to-sleep position (Element 4, < 20%). Other research with pregnant women showed relatively high awareness of fetal movements (84.6%), however, the quality of the information women received lacked consistency [14]. Similarly, findings from a survey of pregnant women in Australia suggest women appreciate the importance of avoiding going-to-sleep on their back in late pregnancy with the most accessed source of information for advice around sleeping position in late pregnancy being their maternity care provider (66%). Despite this, inconsistencies in the information provided to women was common [15].

A similar stillbirth prevention bundle rolled out from 2015 across 19 maternity Trusts in the United Kingdom (UK) demonstrated clear improvements in process outcomes and frequency of performing best practice [16]. Staff views and experiences and women’s experience of care were reported; however, only post-implementation surveys were undertaken and direct comparison with pre-implementation was not possible. Unlike the UK bundle, we had the opportunity to plan the evaluation component of the SBB initiative prior to implementation, this allowed additional opportunities for data collection to inform care and provide a comprehensive view of the impact of implementation.

The aim of this study was to explore the views and experiences of women who had received antenatal care and maternity healthcare professionals (HCPs) who provided antenatal care in relation to the five SBB care elements. Specifically, we sought to assess the impact of SBB implementation on HCP’s reported change in awareness, knowledge, attitudes, and practices; and women’s reported change in awareness and experiences with and quality of antenatal care received related to reducing the risk of stillbirth.

## Methods

### Study design

We undertook a pre-post study of New South Wales (NSW) and Queensland (QLD) maternity services participating in SBB implementation programs (targeted implementers *n* = 61). Surveys were conducted January to July 2020 (pre) and August to December 2022 (post) SBB implementation. Surveys were self-administered using Checkbox (Checkbox Survey Inc., Watertown, MA, USA), an online survey software tool. Responses from the surveys were extracted and imported into Microsoft Excel.

Human research ethics approval was obtained from the Royal Brisbane & Women’s Hospital Human Research Ethics Committee (HREC) in June 2019 (approval number: HREC/2019/QRBW/47,709). The National Health and Medical Research Council have certified the procedures used by this HREC to review multi-centre research proposals. The study protocol outlining the planned evaluation of the SBB across maternity services in New South Wales (NSW), Queensland (QLD) and Victoria (VIC) has been published [10].

### Safer Baby Bundle implementation

The approach to active implementation of the SBB through targeted state-led programs has previously been published [2, 10]. Establishing a dedicated implementation (quality improvement) project team for each state, led by health service executive leadership teams, was a central approach to optimise the reach and uptake of the SBB. The NSW and QLD state project teams supported practice change through co-ordination of education, audit and feedback; and implementation support workshops (learning sessions) to facilitate sharing of knowledge. Local site implementation teams, led by SBB clinical champions, were responsible for roll out at their services.

Implementation and uptake of the SBB was facilitated by a suite of freely available resources providing consistent information for both women and HCPs. These resources were developed by the Stillbirth CRE in partnership with key stakeholders using experience-based co-design methods. Resources developed for clinicians to guide best practice clinical care included position statements (evidence summaries), clinical care pathways, workshops, webinars, masterclasses (for in-service education), and the eLearning package. Corresponding parent-facing resources with key messaging included flyers for each element (translated into 27 languages), fact sheets, waiting room posters, social media tiles and a website. Distribution of these resources was supported by websites managed by the Stillbirth CRE [17, 18], linkages with NSW and QLD Departments of Health websites, and a social media campaign. Prior to the SBB implementation only the DFM element specific brochure for women was widely available in services through the Movements Matter campaign [19] and the My Baby’s Movements trial [20]. Thus, questions around provision of the other element specific brochures were only included in post-SBB implementation surveys.

### Study instruments

Development of the pre/post surveys for women and HCPs and methods of survey administration are described in the study protocol [10] and outlined in brief here. The surveys were developed for this study and drew from the UK bundle evaluation [21].

#### Survey of women

The SBB survey for women who had received antenatal care included demographic characteristics and questions related to experiences of the care they received, awareness and satisfaction with information about stillbirth and reducing their risk of stillbirth. Questions were largely multiple choice with Likert scales. Open text fields followed some multiple-choice questions and invited respondents to expand on a topic. The post-SBB survey included additional questions (*n* = 5) around awareness of the SBB initiative and resources and is provided in [Additional file 1.]

The survey was administered to women following the birth (before hospital discharge or within 6 months of birth). Participation was voluntary and consent was implied if the survey was completed and submitted.

#### Survey of healthcare professionals

To determine attitudes, knowledge and practices around the SBB, HCPs (midwives and doctors) providing antenatal care were invited to complete the pre- and post-implementation surveys. Questions included; the frequency of performing best practice recommendations; satisfaction with resources and training; and attitudes and confidence for talking with women about each element of care. Likert scales for best practice frequency were similar to those from a survey of Australian maternity services undertaken in 2018 during the development of the SBB [11]. The post- SBB survey had additional questions (*n* = 7) around awareness, impressions, and experiences with the SBB initiative and associated resources and is provided in [Additional file 2.]

SBB clinical champions invited HCPs providing antenatal care at their service to undertake surveys. Participation was voluntary and as with women, completion of the survey implied consent. Recruitment was managed within each maternity service and the number of women and HCPs approached to participate is unknown.

### Data management and analysis

The statistical analysis tool used was Stata 17.1 (Stata Corp, College Station, TX, USA) and a *p*-value < 0.05 was considered statistically significant. Microsoft Excel was used to manage open text responses.

Categorical variables were described using frequency (percent) and variables measured on a continuous scale were described using median (interquartile range). For HCPs, 5-point Likert items for frequency of best practice for the five SBB elements were dichotomised for pre-post analysis as ‘All of the time’ and ‘Not all the time’ (most of the time/half of the time/not much of the time/never). Women’s responses for receiving best practice recommendations and information were dichotomised for pre-post analysis as ‘Yes’ and ‘No’ (No/Don’t remember/Unsure) or ‘Yes (at all antenatal appointments from 28 weeks)’ and ‘Not at all antenatal appointments from 28 weeks (No/ Yes (at some appointments)/don’t remember). Other 5-point Likert scale items were collapsed to 3 categories as follows; for level of satisfaction- Unsatisfied (very unsatisfied/unsatisfied), Neutral, Satisfied (satisfied/very satisfied); level of agreement- Disagree (strongly disagree/disagree), Neutral, Agree (strongly agree/agree); and impressions- Negative (very negative/negative), Neutral, Positive (positive/very positive). A key outcome was frequency of HCPs performing best practice recommendations ‘all the time’. Evidence for a difference in perceived practice, knowledge, and confidence between pre- and post- surveys was tested using Pearson’s chi-squared test or Fisher’s exact test for categorical variables and the Wilcoxon rank-sum test for continuous variables. For each variable, missing responses were excluded from analysis and the number of missing responses was reported.

For women’s responses to ‘what was your main model of antenatal care’ those identifying midwifery models of care in pre-defined responses or ‘other’ (open text) were collated and included; midwifery continuity of care, private midwifery, midwifery caseload, midwifery group/team practice and midwifery group practice.

A qualitative content analysis approach guided the analysis of open text responses [22]. Two co-authors (CA, AP) read the text independently to familiarise themselves with responses; ordered data into meaningful groups; looked for re-occurring patterns; and then reviewed and refined these before agreeing on a set of categories that captured the content of responses.

## Results

A total of 1,415 HCPs (pre-1148/post-267) and 1,225 women (pre-1096/post-129) completed surveys. Surveys were completed across the majority of QLD and NSW SBB sites, for HCP pre [61, (100%)] and post [43, (70%)] and women pre [59, (97%)] and post [31, (51%)]. The completion numbers of the post-SBB surveys were substantially lower than pre-SBB. There was a higher percentage of responses from QLD for the HCPs post- SBB surveys compared to the pre-SBB (49.9% pre to 74.9% post, *p* < 0.001), where state representation was more equal. The characteristics of respondents are provided in Table 1. In both pre and post-implementation surveys the majority of responses from HCPs were midwives (pre-82.9%/post-91.0%).

**Table 1:** Characteristics of maternity healthcare professionals (HCP) and women who completed pre/post- SBB surveys

Maternity Healthcare Professionals				
		Pre-SBB *N* = 1,148 *n* (%)	Post-SBB *N* = 267 *n* (%)	*p*-value
State	Queensland	573 (49.9%)	200 (74.9%)	< 0.001^†^
	New South Wales	575 (50.1%)	67 (25.1%)	
Discipline	Midwifery	952 (82.9%)	243 (91.0%)	< 0.001^‡^
	Obstetrics	150 (13.1%)	8 (3.0%)	
	GP	19 (1.7%)	1 (0.4%)	
	Student	20 (1.7%)	7 (2.6%)	
	Other	7 (0.6%)	8 (3.0%)	
Years of experience	Student in training	34 (3.0%)	8 (3.0%)	0.27^†^
	< 5 years	282 (24.6%)	79 (29.6%)	
	5–10 years	232 (20.2%)	57 (21.3%)	
	> 10 years	600 (52.3%)	123 (46.1%)	
Maternity service type	Public hospital only	988 (86.1%)	259 (97.0%)	< 0.001^‡^
	Private hospital only	2 (0.2%)	0 (0.0%)	
	Both public and private	146 (12.7%)	7 (2.6%)	
	Other	12 (1.0%)	1 (0.4%)	

Women				
		Pre-SBB *N* = 1096 n (%)	Post-SBB *N* = 129 n (%)	*p*-value
State	Queensland	356 (32.5%)	74 (57.4%)	< 0.001^†^
	New South Wales	740 (67.5%)	55 (42.6%)	
Country of birth is Australia	Yes	738 (67.3%)*	85 (66.9%)*	0.93^†^
Aboriginal and/or Torres Strait Islander	Yes	60 (5.5%)	7 (5.5%)*	0.99^†^
Age	Less than 18 years	4 (0.4%)	0 (0.0%)	0.32^‡^
	18 to 24 years	173 (15.9%)	21 (16.5%)	
	25 to 34 years	716 (65.6%)	75 (59.1%)	
	35 years or more	198 (18.1%)	31 (24.4%)	
English as first language	Yes	834 (76.1%)	96 (75.6%)*	0.90^†^
Model of antenatal care	Public hospital	728 (66.4%)	84 (65.1%)	0.28^‡^
	Private obstetrician	31 (2.8%)	3 (2.3%)	
	Midwifery	238 (21.7%)	26 (20.2%)	
	GP shared care	96 (8.8%)	14 (10.9%)	
	Other	3 (0.3%)	2 (1.6%)	
Baby’s gestational age at birth (weeks)		39 (38–40)*	39 (38–40)*	0.24^#^
Previous pregnancy	Yes	698 (64.7%)*	69 (54.3%)*	0.022^†^

Statistical tests: ^†^ Pearson’s chi-squared test, ^‡^ Fisher’s exact test, ^#^ Wilcoxon rank-sum. Missing data: * Country of birth is Australia *n* = 2 (post); Aboriginal and/or Torres Strait Islander *n* = 2 (post); English as first language *n* = 2 (post); Gestational age at birth *n* = 107 (pre) *n* = 3 (post); Previous pregnancy *n* = 17 (pre), *n* = 2 (post)

### Provision of best practice recommendations and information related to stillbirth risk and five SBB elements

#### Frequency of HCPs performing best practice recommendations ‘all the time’

The frequency of HCPs performing best practice recommendations ‘all the time’ improved in the post-SBB period across all five elements (Table 2). For pre/post comparison 5-point Likert scales for frequency of performing best practice were collapsed and results for all responses separately is provided [see Additional file 3].

**Table 2:** Comparison pre/post SBB implementation for provision of best practice recommendations and information related to stillbirth risk and SBB elements

Recommendation	Source	Response	Pre *n* (%)	Post *n* (%)	*p*-value^†^
**Element 1- Smoking Cessation**					
Record smoking status at first antenatal visit	HCP	All of the time	836 (85.9%)	197 (89.5%)	0.15
Asked at booking appointment whether you smoked	Women	Yes	985 (89.9%)	112 (86.8%)	0.28
Provide advice on benefits of quitting	HCP	All of the time	582 (54.5%)	172 (74.5%)	< 0.001
Offer personalised advice on how to stop smoking	HCP	All of the time	294 (27.9%)	92 (39.8%)	< 0.001
Refer to Quitline or other stop smoking service	HCP	All of the time	388 (36.6%)	114 (50.0%)	< 0.001
Record passive smoking status at first antenatal visit	HCP	All of the time	447 (46.5%)	124 (56.6%)	0.007
Asked at booking whether regularly exposed to passive smoke	Women	Yes		92 (71.3%)	
Refer partner to Quitline/other if they smoke	HCP	All of the time	160 (15.3%)	55 (24.2%)	0.001
Ask women if they attended Quitline/other appointment	HCP	All of the time	139 (14.0%)	51 (23.1%)	< 0.001
Use ‘Ask, Advise and Help’ brief advice model at every visit	HCP	All of the time	152 (15.6%)	45 (20.3%)	0.088
Offer all women exhaled breath CO reading	HCP	All of the time	38 (4.5%)	14 (8.3%)	0.042
CO Breath test offered	Women	Yes	26 (2.4%)	3 (2.3%)	1.00^‡^
Quit smoking brochure provided	HCP	Yes		170 (72.3%)	
Quit smoking brochure received and read	Women	Yes		58 (45.0%)	
**Element 2- Fetal Growth Restriction (FGR)**					
Assess for risk factors for FGR early in pregnancy	HCP	All of the time	597 (59.2%)	190 (84.1%)	< 0.001
Assess for risk factors for FGR at visits from 24 weeks’	HCP	All of the time	630 (61.2%)	165 (72.4%)	0.002
SFH measure at visits from 24 weeks’	HCP	All of the time	875 (85.0%)	207 (90.4%)	0.035
SFH measured (at all antenatal appointments from 28 weeks’)	Woman	Yes	939 (85.7%)	105 (81.4%)	0.20
Plot SFH on growth chart	HCP	All of the time	228 (22.8%)	110 (48.7%)	< 0.001
Refer for growth scans if at increased risk	HCP	All of the time	481 (51.3%)	137 (69.5%)	< 0.001
Growth Matters brochure provided	HCP	Yes		124 (53.4%)	
Growth Matters brochure received and read	Women	Yes		63 (48.8%)	
**Element 3- Decreased Fetal Movements**					
Discuss importance of reporting DFM, each visit from 28 weeks’	HCP	All of the time	884 (83.3%)	228 (93.1%)	< 0.001
Baby’s movements discussed, each visit from 28 weeks’	Women	Yes	752 (68.6%)	107 (82.9%)	< 0.001
From 28 weeks’, how often CTG within 2 h if concern about DFM	HCP	All of the time	869 (79.9%)	198 (81.8%)	0.51
Movements Matter brochure provided	HCP	Yes	496 (43.2%)	206 (85.1%)	< 0.001
Movements Matter brochure received and read	Women	Yes	454 (41.4%)	95 (73.6%)	< 0.001
**Element 4- Maternal Safe Sleeping Position**					
Provide information and discuss safe sleep position by 28 weeks’	HCP	All of the time	216 (20.4%)	193 (79.4%)	< 0.001
Discuss safe going-to-sleep position at every visit from 28 weeks’	HCP	All of the time	241 (22.8%)	164 (66.1%)	< 0.001
Importance of sleeping on side in late pregnancy discussed (at all antenatal appointments from 28 weeks’)	Women	Yes	285 (26.0%)	71 (55.0%)	< 0.001
Sleep-on-side brochure provided	HCP	Yes		202 (81.8%)	
Sleep-on-side brochure received and read	Women	Yes		104 (80.6%)	
**Element 5- Timing of Birth**					
Assess for stillbirth risk factors first antenatal visit	HCP	All of the time	436 (44.5%)	167 (73.2%)	< 0.001
Reassess for stillbirth risk factors 34 to 36 + 6 weeks gestation	HCP	All of the time	265 (26.3%)	110 (47.2%)	< 0.001
Discuss birth planning according to risk status	HCP	All of the time	296 (29.1%)	108 (46.0%)	< 0.001
Possibility of having a planned birth discussed	Women	Yes	545 (49.7%)	81 (62.8%)	0.005
Provide individual information about birth timing based on stillbirth risk	HCP	All of the time	303 (29.8%)	138 (57.5%)	< 0.001
Involved as much as you wanted to be when making decisions and choosing options about the timing of your baby’s birth	Women	Yes	825 (75.3%)	97 (75.2%)	0.98

For pre/post analysis, HCPs frequency of best practice were dichotomised as ‘All of the time’ and ‘Not all the time’ (most of the time/half of the time/not much of the time/never), Women’s responses were dichotomised as ‘Yes’ and ‘No’ (No/Don’t remember/Unsure) or ‘Yes (at all antenatal appointments from 28 weeks)’ and ‘Not at all antenatal appointments from 28 weeks (No/ Yes (at some appointments)/don’t remember). HCP- Healthcare professional, CO- Carbon Monoxide, SFH- Symphyseal Fundal Height, FGR- Fetal Growth Restriction, DFM- Decreased Fetal Movements, CTG- Cardiotocography, ^†^ Pearson’s chi-squared test, ^‡^ Fisher’s exact test

#### Providing and receiving information around the five SBB elements

Provision of information and conversations around the five SBB elements improved post-implementation. The magnitude of change varied and, as anticipated, was smallest where baseline levels (pre-SBB) were highest. Alignment between HCPs self-reported provision of information and women reporting receiving and reading information was consistent, with the most variation seen for provision of Quit smoking brochure (72.3% of clinicians providing the brochure, only 45.0% of women reporting receiving and reading the brochure). However, some of this difference may be accounted for by an additional 20% of women who reported receiving the brochure, but not reading it. See Table 2 and Additional file 3. SBB element specific findings include:

- Element 1: HCPs reported provision of advice on the benefits of quitting smoking increased (54.5–74.5%, *p* < 0.001). A high proportion of women reported being asked at booking about their smoking status pre- SBB, this remained high (89.9–86.8%, *p* = 0.28). In the post-SBB period only half of HCPs surveyed reported referring smokers to Quitline or other stop smoking services, showing a small improvement (36.6–50.0%, < 0.001). Additionally, uptake of the ‘Ask, Advise and Help’ brief advice model at each visit was low and did not improve significantly (15.6–20.3%, *p* = 0.088). Similarly low was uptake of offering exhaled breath CO testing (4.5–8.5%).
- Element 2: Routine measuring of Symphyseal Fundal Height (SFH) as reported by HCPs was high pre- SBB and further improved post (85.0–90.4%, *p* < 0.035). This aligns with most women recalling their ‘tummy being measured for baby’s growth’ at all antenatal appointments from 28wks (85.7–81.4%, *p* = 0.20), although an increase was not observed in the post- SBB period.
- Element 3: Post-SBB implementation more women received and read the DFM brochure (41.4–73.6%, *p* < 0.001). Nearly double the number of HCPs reported routinely providing the DFM brochure to women post- SBB (43.2–85.1%, *p* < 0.001).
- Element 4: Post- SBB HCPs reported reliability of provision of information and discussing safe sleep position by 28 weeks improved considerably (20.4–79.4%, *p* < 0.001). Correspondingly more women recalled the importance of sleeping on their side in late pregnancy being discussed at all antenatal appointments from 28 weeks (26.0–55.0%, *p* < 0.001) and post-SBB implementation the majority (80.6%) recalled receiving and reading the sleep-on-side brochure.
- Element 5: An increase in HCPs reporting discussing timing of birth planning ‘all the time’ was seen (29.1–46.0%, *p* < 0.001). This is consistent with an increase in the proportion of women who recall discussing the possibility of birth timing plans (49.7–62.8%, *p* = 0.005). Three out of four women reported being involved as much as they wanted to be when making decisions about the timing of their baby’s birth (75.3–75.2%, *p* = 0.98) and this did not change post-SBB implementation.

### Conversations about stillbirth prevention and risk of stillbirth

**Fig. 2:**
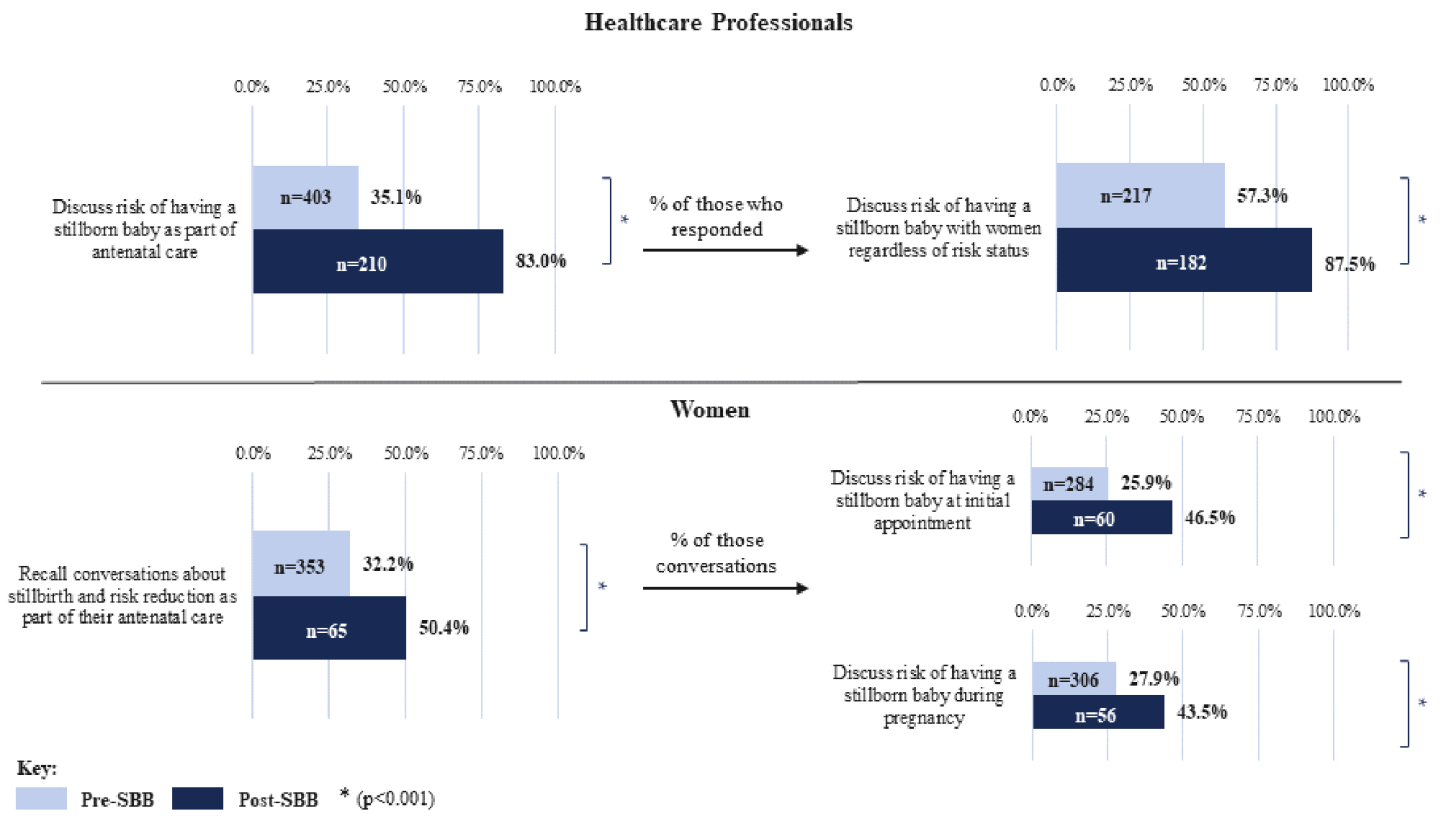
Conversations about stillbirth and risk reduction pre/post- SBB implementation. *p < 0.001

Post-SBB implementation, more women recalled conversations about stillbirth and risk reduction as part of their antenatal care (32.2–50.4%, *p* < 0.001), Fig. 2. Improvement for the percentage of HCPs indicating they include conversations about stillbirth as routine antenatal care (35.1–83.0%, *p* < 0.001) was greater. However, some difference may be accounted for with approximately one in five women responding either they ‘don’t ‘remember’ or were ‘unsure (as not sure what the risk factors are)’. Of those HCPs who responded ‘yes’, they discuss stillbirth risk as part of their antenatal care, in the post-SBB implementation period a greater proportion reported having these conversations regardless of a woman’s risk status (57.3–87.5%, *p* < 0.001). Post- SBB implementation there was also a trend towards having this conversation earlier in pregnancy shown as an increase in HCPs reporting first having this discussion in the first trimester (7.5–15.5%) and second trimester (47.4–59.1%) with less in the third trimester (45.1–25.4%).

#### Women’s perceptions of conversations around stillbirth and risk factors for stillbirth

Several thematic categories were identified through analysis of open text responses related to women’s’ feelings towards the care and information they received during pregnancy about stillbirth and risk factors for stillbirth and/or planning the timing of their baby’s birth. There were 248 responses for analysis (pre-210/post-38). Four main categories were similar across pre-post responses and identified as follows:

- **Informed and reassured**- These responses from women indicated a positive attitude towards receiving information and the value of being informed.
  - *‘Informed. Happy for it to be discussed I liked to know.’*; ‘*I personally like any and all information given to me regardless of the topic*,* need to be informed at all times.’;* ‘*I was greatly reassured that I had some choices available around birth due to my anxiety about pregnancy loss.’; ‘Care was very informative and caring.’.*
- **Overwhelmed and confronted**- These responses indicated a negative attitude towards receiving information, suggesting for some women these conversations may increase anxiety and/or be perceived as insensitive.
  - ‘*It made me worry too much’; ‘I felt a bit nervous’; ‘overwhelmed’; ‘I didn’t want to hear it*,* unnecessary’*; *‘Was very abrupt and it was quite confronting as I’d never known that was a risk’*.
- **Silence about stillbirth and risk factors-** Several responses signalled a silence about stillbirth and risk factors, with women not recalling this being mentioned or discussed at all during their antenatal care.
  - ‘*The possibility of stillbirth was never really mentioned by any provider’; ‘I was induced at 39 weeks due to gestational diabetes*,* the risk of stillbirth was never discussed with me at any point.’*
- **Choices not sufficiently informed and/or respected-** In relation to planning the timing of their baby’s birth, some responses suggested women felt as though they were not provided with sufficient information to inform decisions about their care. A few responses also suggested women did not feel heard or respected when making choices about their care.
  - *'I wish that we were kept informed more. Since our situation was changing week to week no one had informed us that our choices changed as well.’; ‘I would like to see more discussion from doctors regarding possible risks when I was contacted to request I be induced.’; ‘A midwife I had was quite stern and I felt like I couldn’t stand up to her and say I wasn’t happy with the way she wanted to do things’; ‘Did not feel as though I was involved in the decision making and was questioned multiple times regarding my choice*,* without reasons or explanations’.*

### Healthcare professionals change in knowledge and confidence

HCPs confidence in their level of knowledge and comfort when thinking about having a conversation with women about the five SBB elements was most improved for timing of birth (49.5–85.4%, *p* < 0.001) and FGR (59.4–90.5%, *p* < 0.001) [see Additional file 4.] Many HCPs were concerned that conversations across all SBB elements may cause anxiety for women (range 21.0 − 52.9% (pre) to 13.5 − 56.5% (post)). Conversations around safe maternal sleep position was the element for which there was the lowest concerns at baseline, and post-SBB implementation (21.0–13.5%, *p* < 0.001). In the post-SBB implementation period, across all five elements fewer HCPs feel having these conversations would negatively impact on their relationship with women (range 5.1–25.2% (pre) to 3.0–18.3% (post)). However, for conversations around smoking cessation, although gains are demonstrated (25.2–18.3%, *p* = 0.020), nearly one in five HCPs remain concerned about having these conversations.

### Safer Baby Bundle awareness and impact (Post-SBB implementation survey only)

#### Healthcare professionals post-SBB implementation

**Fig. 3:**
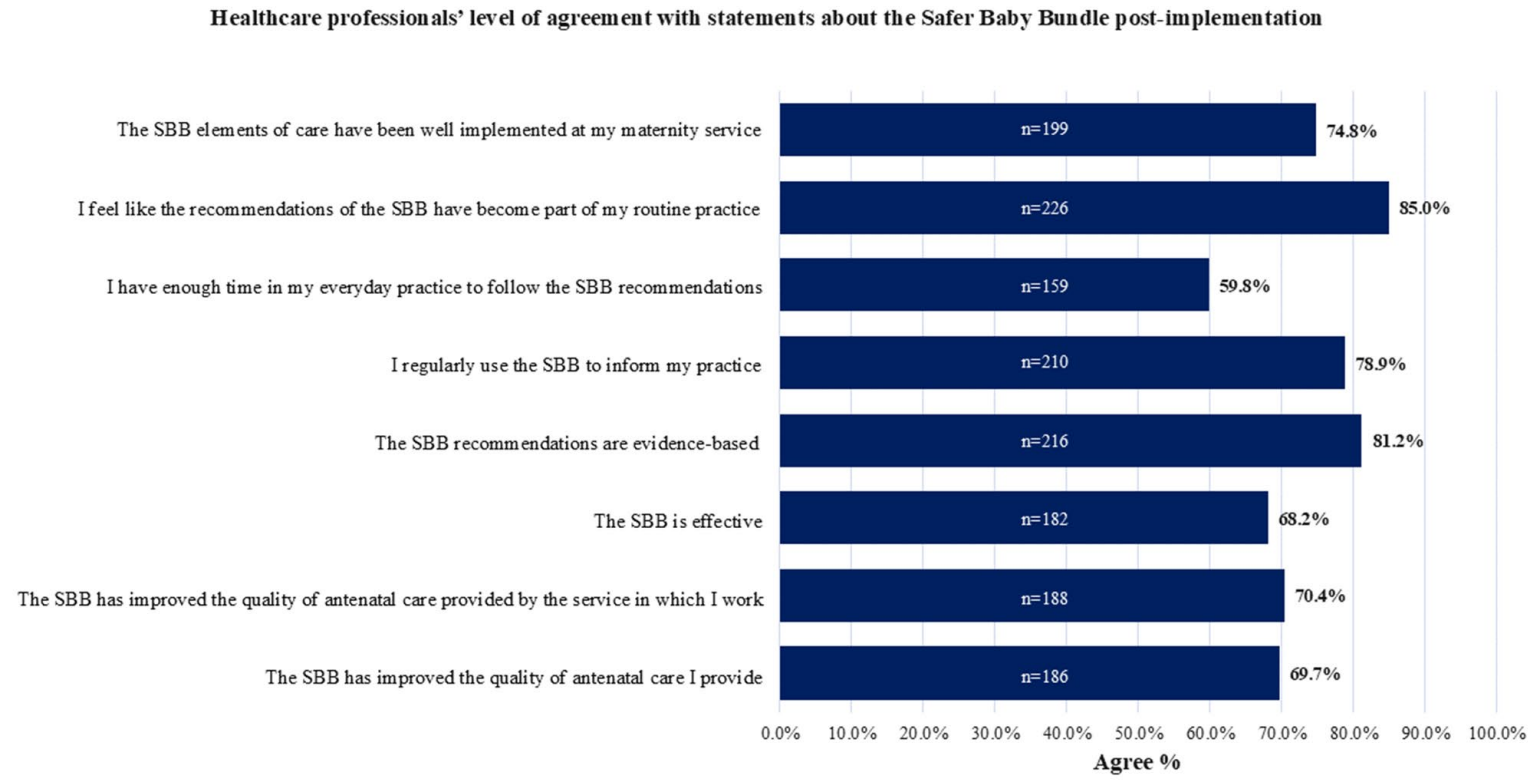
Healthcare professionals’ level of agreement (agree = strongly agree/agree) with statements considering the SBB initiative post-implementation

Post-SBB nearly all HCPs surveyed [260, (97.4%)] had heard about the SBB. Of those, 99.2% (258) were aware that the SBB has been implemented at their service. HCPs were first made aware of the SBB through peer-to-peer communication (28%), in-service education (26%), and eLearning (25%). Overall, approximately 3 out of 4 HCP perceived the impact of implementing the SBB elements at their service as positive [202 (75.7%)], [see Additional file 5]. However, only just over half [159 (59.8%)] agreed to having enough time to follow the SBB recommendations in their everyday practice. Most [226 (85.0%)] agreed that the recommendations of the SBB had become part of their routine practice and the majority agreed that the SBB had been well implemented at their service [199 (74.8%)] and has improved the quality of antenatal care they [186 (69.7%)] and their maternity service [188 (70.4%)] provide, see Fig. 3.

#### Women receiving antenatal care

Post-SBB implementation most HCPs [231 (86.5%)] indicated they provide women with access to the SBB resources, with half of the women [71 (55.0%)] reporting reading the Safer Baby brochure, and less than half [59 (45.7%)] indicating they were aware of the SBB prior to undertaking the survey.

### Satisfaction with antenatal care and information

Most women surveyed remained satisfied (satisfied/very satisfied) across all domains relating to information and care provided during pregnancy in general: information about choices for maternity care (76.2–75.2%), information to help decide about care (76.6–76.7%), given information at the right time (76.6–79.8%), and having confidence and trust in the staff caring for you (89.5–87.6%).

HCPs level of satisfaction with the support and information provided to women attending their services improved significantly (*p* < 0.001) across all 5 elements. The magnitude of improvement was greatest for elements with a lower baseline satisfaction such as side sleeping (33.9–89.8%, Element 4), TOB (35.8–69.6%, Element 5) and smoking cessation (38.1–67.5%, Element 1). Whilst the magnitude of change was less, satisfaction post- SBB implementation was high for FGR (57.4–77.4%, Element 2) and DFM (72.3–86.8%, Element 3).

### Adequacy of HCP training

**Fig. 4:**
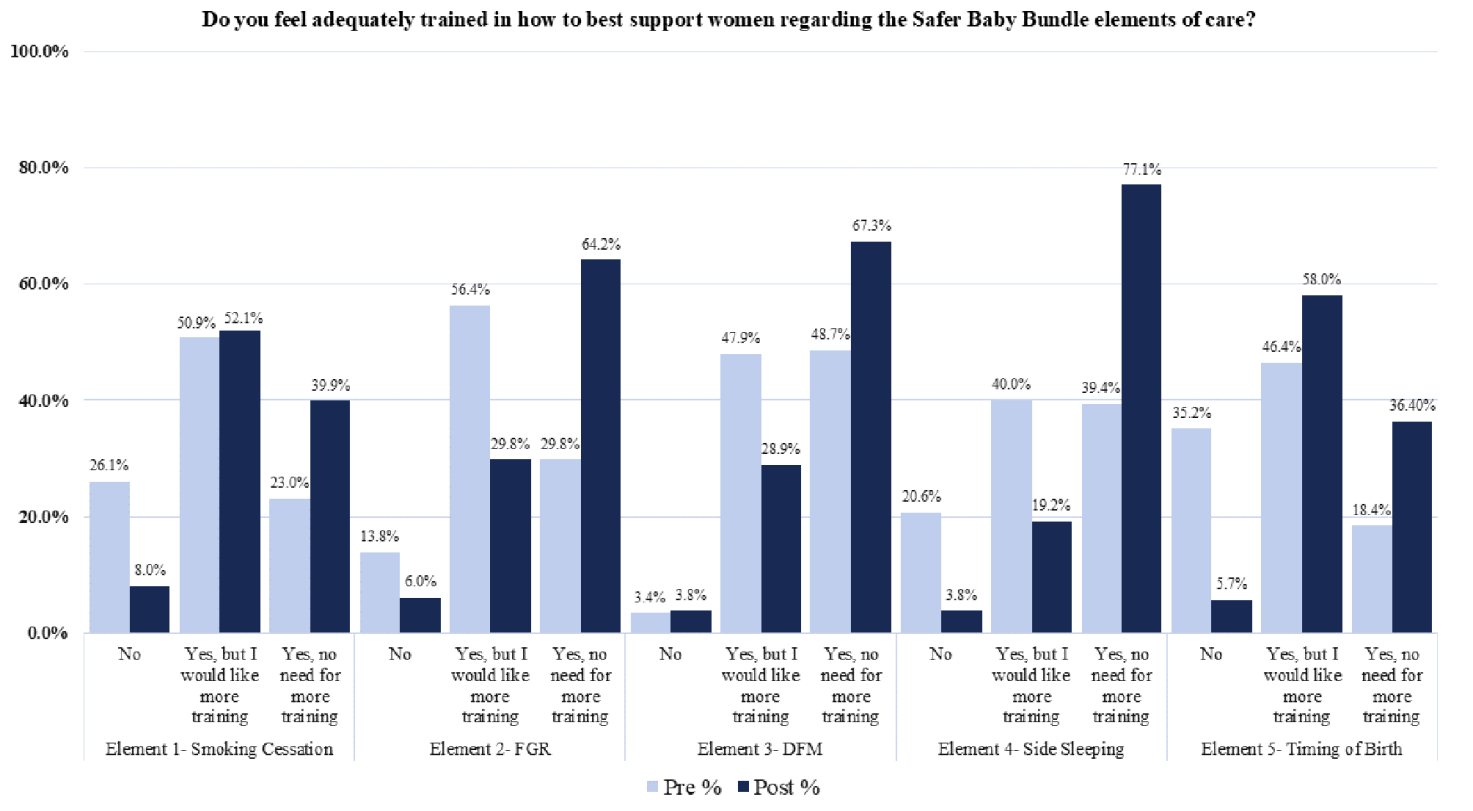
Comparing adequacy of healthcare professionals’ training across the five elements pre/post- SBB implementation

Across all five SBB elements post- SBB implementation, HCPs were more likely to report that they were ‘adequately trained, with no need for more training’ (*p* < 0.001), see Fig. 4. Post- SBB implementation, a small number of HCPs indicated they did not feel adequately trained, this was highest for smoking cessation (Element 1–8.0%), FGR (Element 2–6.0%) and timing of birth (Element 5–5.7%).

## Discussion

This pre-post SBB implementation survey analysis found that self-reported experiences of providing and receiving antenatal care in relation to reducing the risk of stillbirth substantively improved across all five elements. Positive changes in HCPs awareness, knowledge and practices were seen including an increase in; provision of advice on the benefits of quitting smoking; assessing risk factors and surveillance for growth restriction in early pregnancy; provision of information and advice regarding maternal safe sleeping position; and discussing the importance of monitoring fetal movements and reporting concerns. Improvements were accompanied by HCPs self-reported increased confidence in their level of knowledge across all five elements. Reassuringly, key findings from women who received antenatal care at services which had implemented the SBB were complementary to this finding. After SBB implementation, the proportion of women recalling being informed about stillbirth risk reduction strategies during their antenatal care was nearly double.

The magnitude of improvements shown varied by element, with reliability of provision of information and advice most increased for maternal going-to-sleep position (Element 4). In Australia, prior to the SBB there had been limited efforts aimed at improving awareness and education across maternal safe sleeping position, which may explain why improvements shown were the largest and most consistent. However, it is probable that higher uptake of recommendations for this element were achieved as HCPs perceived these conversations as least likely to cause anxiety or negatively impact on their relationship with the woman. Conversely, whilst improvements shown across smoking cessation (Element 1) recommendations are encouraging, uptake was more varied. Conversations surrounding smoking cessation in pregnancy are seen as difficult, with HCPs often feeling concerned they may harm their relationship with the women and/or deter future attendance for antenatal care [23–25]. Similar barriers are apparent in this study, with nearly one in five HCPs in the post- SBB period remaining concerned these conversations may negatively impact their relationships with women and half indicating that they would still like more training. Midwives are pivotal in any approach to reduce smoking rates amongst pregnant women [26]. Thus, further specific training showing ‘how to have these conversations’ is needed and must be informed by recognition of the centrality of women/HCPs relationships in all interactions.

Reassuringly significant improvements across DFM (Element 3) were shown, notwithstanding higher baseline levels. Previous research in 2017 had shown that reliability of DFM messaging was inconsistent and potentially perpetuating myths such as baby running out of room and movements slowing down near the end of the pregnancy [14]. Prior to the SBB, efforts in Australia to improve awareness and management of DFM were in progress, supported by a social media and hospital-based awareness campaign in Victoria (Movements Matter [19], 2018) and a large multi-jurisdictional study (My Baby’s Movements [20], 2016 to 2019). The progressive and sustained improvement shown for DFM awareness and management nationally across the last decade, enhanced by the SBB, highlights the necessity for a continued commitment to making improvements and the importance of utilising a multi-layered strategy to influence and sustain behaviour change.

The proportion of HCPs performing assessments for risk factors for both FGR and stillbirth in early pregnancy ‘all the time’ was high in the post-SBB period (84% and 73% respectively). This is essential to enabling HCPs to provide risk-appropriate perinatal care. However, resource implications for some of the care pathways may have been a barrier to consistent implementation for other FGR (Element 2) and timing of birth (Element 5) recommendations. For example, increasing referrals for growth scans for those at increased risk is a complex systems change, requiring multi-disciplinary engagement and additional ultrasound capacity [16]. Similarly, discussing birth planning according to individualised stillbirth risk status is challenging as HCPs often find counselling women about stillbirth risk complex. A pattern for increased uptake of SBB best-practice recommendations was observed where the practice change required is procedural at routine appointments, predominantly involving only midwives and with minimal resource implications. Furthermore, only around 60% of HCPs agreed to feeling they have enough time to follow the SBB recommendations in their everyday practice. Thus, strategies to improve the uptake of best practice that were more resistant to change (such as referral for growth scans and discussing birth planning according to stillbirth risk) need further consideration and are likely to benefit from increased allocation of staff time and resources.

Like the UK bundle [16], in post-SBB implementation 3 out of 4 women reported receiving and reading the DFM brochure [16]. Unlike the UK bundle where 70% of women reported being offered a Carbon Monoxide (CO) test (with 99% accepting the offer) [16], only a very small percentage (2%) of women reported this being offered post-SBB implementation. Use of CO monitors was not standard practice in Australia prior to the SBB, and implementation of this recommendation stalled due to coinciding with the COVID-19 pandemic and jurisdiction infection control guidelines which inhibited their use. Funding for purchase and/or maintenance of CO monitors and consumables was not included within the program’s implementation support and likely further deterred uptake. A systematic review of CO testing in pregnancy found whilst some research suggests their use to be a non-confrontational way to raise the topic of smoking and cessation, barriers to use include time constraints and concern about relationships if testing is not conducted well [27]. Thus, further evidence is needed to support uptake for the recommendation of routine CO monitoring in pregnancy.

Recommendations for Element 5 of the SBB emphasise the importance of involving women in their care and decision-making and reducing unnecessary interventions. Similarly, an updated version of the UK care bundle (Version Two [28]) acknowledges the high importance of ensuring women are involved in their care. Encouragingly, this study provides some evidence that many women receiving antenatal care in Australia are satisfied with their care and their level of involvement, with three out of four reporting being involved as much as they wanted to be when making decisions about the timing of their baby’s birth (both pre- and post- SBB). However, findings should be interpreted cautiously as this is a complex issue which may not be fully appreciated by single survey response and opportunities for improving involvement and respect for informed preferences remain.

High visibility of the SBB initiative with frontline HCPs at participating services was apparent, with almost all having an awareness that it had been implemented at their service. Contrastingly, post-implementation surveys conducted for the UK stillbirth prevention bundle showed 42% of HCPs were unaware the bundle had been implemented. To support implementation many services established the SBB online education program [13] as mandatory training, which, along with strong health executive buy-in, likely contributed to the extensive reach of the initiative. Previously identified core enablers to the provision of best practice included increasing staff awareness and availability of consistent recommendations; and addressing inconsistencies in staff knowledge [11]. The reported findings of improved HCPs knowledge, confidence and perceived adequacy of training related to stillbirth risk and across the five SBB elements are thus foundational to gains seen post-SBB for frequency with which best practice is being performed.

The baseline findings concur with previous reports [14] indicating that prior to the SBB there was a silence around stillbirth during antenatal care, which reduces awareness amongst women about how to minimise their chance of stillbirth. Similarly, studies from Ireland have shown that women with ‘uncomplicated’ pregnancies receive limited information about stillbirth during pregnancy and that most women perceived receiving information about stillbirth during antenatal care to be useful to help preventive efforts [29]. Implementation of the SBB doubled the number of HCPs having conversations and providing written resources around stillbirth risk reduction regardless of women’s risk profiles. This is an important achievement, as increasing awareness amongst women about how to minimise their chance of stillbirth is key to reducing stillbirth rates. This does not alter the understanding that communication about stillbirth and related modifiable factors during pregnancy is difficult for HCPs and women. A systematic review of behaviour change techniques used in the context of stillbirth prevention concluded that these conversations can be uncomfortable or stressful for HCPs [30] and many have concerns these discussions create unnecessary anxiety for pregnant women [14]. Consistent with the literature, our findings show before implementation of the SBB, more HCPs reported feeling concerned these conversations may cause anxiety for women and avoided these discussions. Women’s feelings towards these conversations were mixed, supporting contentions that discussing stillbirth during pregnancy is perceived as a difficult topic [29]. Whilst some women felt there was a lack of discussion about stillbirth, for others a balance between the pros and cons of receiving this information was evident with some feeling informed, reassured, and cared for, whilst others perceived this as unnecessary or worrying. In depth interviews with women and HCPs to further explore experiences and attitudes towards having these conversations have been undertaken and will be reported elsewhere. Findings from the current study highlight the complexities of these conversations and demonstrate the importance of having appropriate co-designed resources and training to support effective communication.

### Strengths and limitations

A strength of this study was inclusion of both women and HCPs experiences to enable a more holistic and robust impression of the impact of SBB implementation on practice change. The results are representative of a large sample of maternity services recruited as ‘targeted implementers’ in NSW and QLD. Findings presented here report on self-reported practices and will be further complemented by process, impact, and clinical outcome evaluations using routinely collected perinatal data, clinical audits and in-depth interviews. The results of these other components will be reported elsewhere.

The large number of completed surveys in the pre-SBB period gives strength to the representativeness and quality of these baseline data. However, the comparatively low survey completion numbers in the post-SBB implementation period, particularly for women and medical staff, was a limitation. This may have increased the likelihood of selection bias (women who had a particularly positive or negative experience might be more likely to have responded to the survey). It is likely the length of the surveys may have impacted the completion rate, however, even taking this into consideration, post-SBB fewer potential participants opened the link to the survey. Several strategies to maximise response rates were employed including multiple recruitment methods and extended data collection periods. Whilst these strategies were successful pre- SBB in early 2020, post-SBB implementation surveys conducted in late 2022 (post-COVID- 19) had a substantively lower response rate. During the pre-SBB implementation period there was considerable buy-in and enthusiasm amongst HCPs and maternity service executives for this large-scale interjurisdictional quality improvement initiative, which likely strengthened participation. The timing of implementation spanned the COVID-19 pandemic leading to extensive delays and disruptions with implementation efforts, which markedly extended planned rollout timelines and strained workforce capacity. Thus, prioritisation, time, and enthusiasm for recruiting to research data collection had waned and was seen as more burdensome for SBB service champions towards the end of the project. Particularly noted for HCPs [31], survey fatigue is also an important factor in the willingness to participate in online surveys. A recently postulated driver of survey fatigue is the overwhelming volume of research undertaken during COVID-19 [32]. Health services and HCPs targeted to implement the SBB are the same workforces that have been severely impacted by COVID-19 related disruptions and increased workloads and their capacity to support research activities (including survey dissemination) was hugely impacted by resource and staff shortages over the study time period.

The surveys were administered within a service quality improvement framework and the invitation to participate was broadly disseminated to women by HCPs as part of post-natal care, thus the number of women approached or who received the survey invitation is unknown and a response rate is not reported. As such, comparison between respondents and nonrespondents was not possible and any possible bias as a result cannot be determined. Administering the survey to women following the birth (before hospital discharge) or within 6 months of birth was needed to support sufficient completions, however, women’s perception of their care may change over time, and this is a limitation. Finally, the magnitude of practice change was self-reported by individual HCPs and may not accurately represent the degree of change within each maternity service.

## Conclusion

This study strongly indicates that implementation of the Safer Baby Bundle in Australian maternity settings results in important improvements in recommended antenatal care practices linked to stillbirth reduction and has been well received by HCPs (particularly midwives) and women. For women, conversations, and provision of information around stillbirth risk reduction during their antenatal care is more consistent. However, women in this study reported varying attitudes regarding provision of information about stillbirth risk and/or planning the timing of their baby’s birth. This included concerns about insufficient information and that choices were not respected. Ongoing research as part of enhancement to the SBB to support shared decision-making for women and HCPs around timing of birth, using an individualised risk factor-based approach, may help to further improve experiences of women in this challenging area.

We anticipate that the positive changes in reported practices shown in this study will translate into improved experiences of care and the targeted reduction in late gestation stillbirth rates in Australia.

## Electronic supplementary material

Below is the link to the electronic supplementary material.

Supplementary Material 1

Supplementary Material 2

Supplementary Material 3

Supplementary Material 4

Supplementary Material 5

## Data Availability

The data that support the findings of this study are available from the corresponding author upon reasonable request. Data are located in controlled access data storage at Mater Research Institute.

## Abbreviations

CEC: Clinical Excellence Commission
CEQ: Clinical Excellence Queensland
CO: Carbon Monoxide
DFM: Decreased fetal movements
FGR: Fetal growth restriction
HCP: Healthcare professional
HREC: Human Research Ethics Committee
ICHOM: International Consortium for Health Outcomes Measurement
NHMRC: National Health and Medical Research Council
NSW: New South Wales
PSANZ: Perinatal Society of Australia and New Zealand
QI: Quality Improvement
QLD: Queensland
SBB: Safer Baby Bundle
SBLCB: Saving Babies Lives Care Bundle
SFH: Symphyseal-fundal height
Stillbirth CRE: The Centre of Research Excellence in Stillbirth
TOB: Timing of birth
UK: United Kingdom
VIC: Victoria
