# Correction: Experiences of antenatal care practices to reduce stillbirth: surveys of women and healthcare professionals pre-post implementation of the Safer Baby Bundle

**DOI:** 10.1186/s12884-024-06776-6

**Published:** 2024-09-28

**Authors:** Christine Andrews, Frances M. Boyle, Ashley Pade, Philippa Middleton, David Ellwood, Adrienne Gordon, Miranda Davies-Tuck, Caroline Homer, Alison Griffin, Michael Nicholl, Kirstine Sketcher-Baker, Vicki Flenady

**Affiliations:** 1 Centre of Research Excellence in Stillbirth, Mater Research Institute, The University of Queensland, Brisbane, Australia; 2 Institute for Social Science Research, The University of Queensland, Brisbane, Australia; 3 South Australian Health and Medical Research Institute, Adelaide, Australia; 4 School of Medicine and Dentistry, Griffith University, Queensland, Australia; 5 School of Medicine, The University of Sydney, Sydney, Australia; 6 The Ritchie Centre, Hudson Institute of Medical Research, Melbourne, Australia; 7 Department of Obstetrics and Gynaecology, Monash University, Melbourne, Australia; 8 Burnet Institute, Melbourne, Australia; 9 QIMR Berghofer Medical Research Institute, Brisbane, Australia; 10 Clinical Excellence Commission, NSW Health, Sydney, Australia; 11 Clinical Excellence Queensland, Brisbane, Australia; 12 The University of Adelaide, Adelaide, Australia; 13 Gold Coast University Hospital, Gold Coast, QLD Australia

**Correction: BMC Pregnancy Childbirth 24**,** 520 (2024)**

10.1186/s12884-024-06712-8

Following publication of the original article [1], the authors reported an error in Fig. 2. The colours in the key representing the pre/post bars in the graph for Fig. 2. are incorrect (around the wrong way). This relates to the pre/post-intervention findings and it is very important for this to be corrected.

The correct figure is given below.

**Fig. 2:**
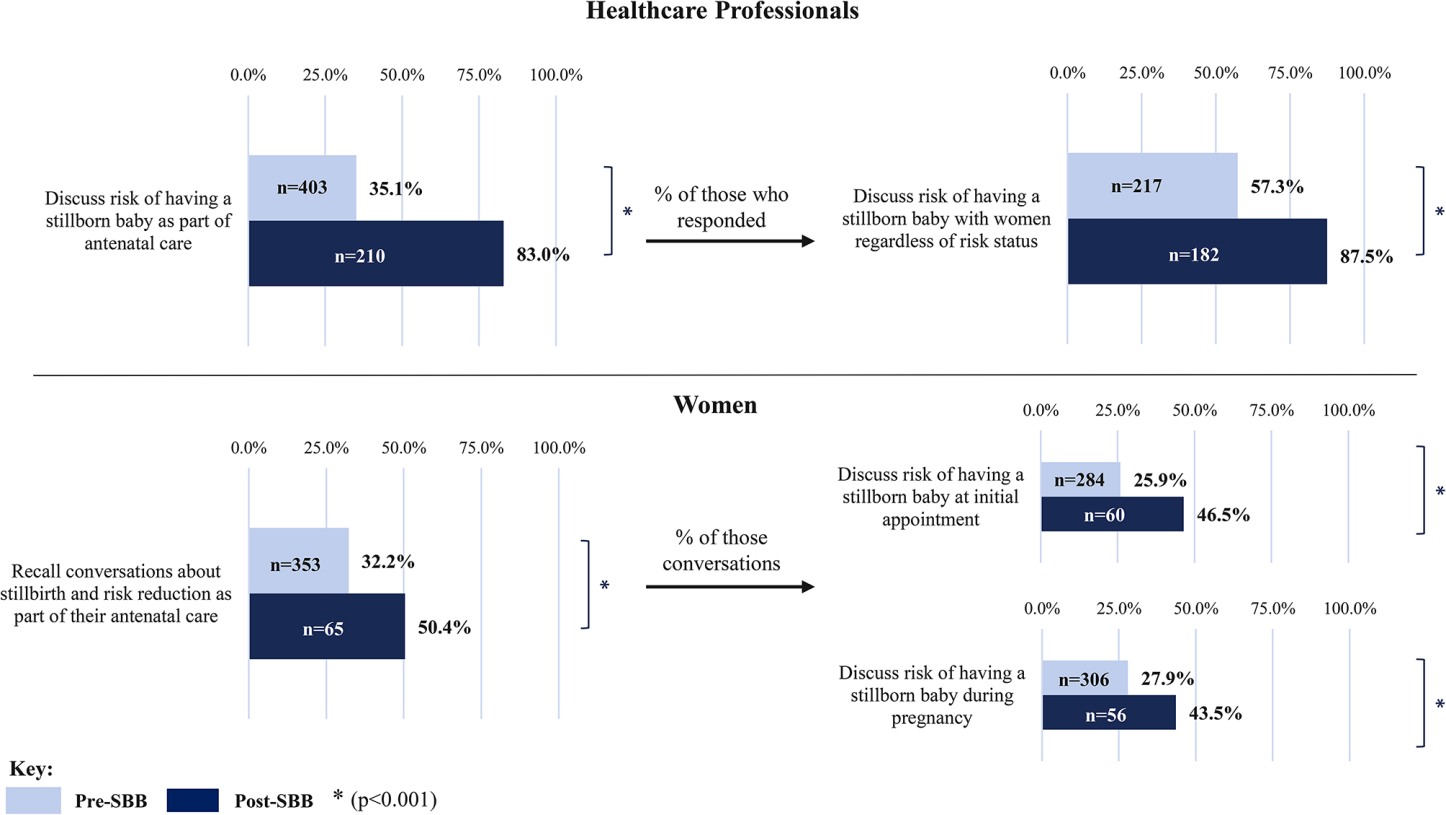
Conversations about stillbirth and risk reduction pre/post- SBB implementation. *p < 0.001

The original article has been corrected.
