# Supplementary material for: Experiences of antenatal care practices to reduce stillbirth: surveys of women and healthcare professionals pre-post implementation of the Safer Baby Bundle
Source: BMC Pregnancy Childbirth. 2024 Aug 1;24:520. doi: 10.1186/s12884-024-06712-8 (PMC11295589; doi:10.1186/s12884-024-06712-8)
Supplement: Supplementary file 1 — Supplementary Material 1 [file 12884_2024_6712_MOESM1_ESM.docx]

**Additional file 1: Women’s online survey tool**

**Survey about experiences of care during pregnancy – Post implementation of the Safer Baby Bundle**

**Section 1: Experience with antenatal care**

1. What was your main model of antenatal care? *(Multiple choice)*
2. Public hospital care
3. Private obstetrician
4. Private midwifery
5. General practitioner shared care
6. Midwifery group practice
7. Midwifery continuity of care Unsure
8. Other:
9. Thinking about your care during your pregnancy: *(Matrix, multiple choice)*

|  | No | To some extent | Yes |
| --- | --- | --- | --- |
| Were you given information about your choices for maternity care? |  |  |  |
| Were you given enough information to help you decide about your care? |  |  |  |
| Were you given information at the right time to help you decide about your care? |  |  |  |
| Did you have confidence and trust in the staff caring for you? |  |  |  |

1. How satisfied are you with the care you received during your pregnancy?
2. Very Unsatisfied
3. Unsatisfied
4. Neither satisfied nor dissatisfied
5. Satisfied
6. Very Satisfied

- What mattered most to you during your antenatal appointments? (Open text, optional question)

**Section 2: Safer Baby resources**

1. ***POST IMPLEMENTATION SURVEY QUESTION ONLY*** Were you given/have you seen any of the following brochures? (Matrix)

|  | Yes, I read the brochure | Yes, but I did not read the brochure | No, but I was given a different brochure on this topic | No, I was not given a brochure on this topic | Don’t remember |
| --- | --- | --- | --- | --- | --- |
| Quit smoking for baby brochure |  |  |  |  |  |
| Your baby’s growth matters brochure |  |  |  |  |  |
| Your baby’s movements matter brochure |  |  |  |  |  |
| Sleep on your side brochure |  |  |  |  |  |
| Safer Baby brochure |  |  |  |  |  |

- If you were given a different resource or brochure for any of the above elements of care (topics), can you please tell us about these? *(Open text, optional question)*

1. ***POST IMPLEMENTATION SURVEY QUESTION ONLY*** Prior to this survey, were you aware of the Safer Baby initiative? *(Multiple choice)*
2. Yes
3. No (skip to Q7)
4. Not sure (skip to Q7)
5. ***POST IMPLEMENTATION SURVEY QUESTION ONLY*** How did you mainly hear about the Safer Baby initiative? *(Checkbox- select all that apply)*
6. At the doctors
7. At the hospital (or antenatal clinic)
8. TV
9. Radio
10. Newspaper/magazine
11. Website
12. Social media
13. Other:
14. ***POST IMPLEMENTATION SURVEY QUESTION ONLY*** Have you seen or heard any of the following messages about stillbirth in the media or on line? Please select all that apply. *(Checkbox- select all that apply)*
15. A couple sharing their story of having a stillborn baby
16. Make the Stillbirth Promise
17. Quit for baby, stop smoking during pregnancy
18. Big or small. Your baby's growth matters
19. Be aware of your baby's movements
20. Get to know your baby's normal
21. Sleep on your side after 28 weeks
22. Stillbirth. Together we can reduce the risk
23. Six babies are stillborn everyday
24. Don't know/None of the above

**Section 3: Element 1 (smoking cessation)**

1. Did you smoke at the beginning of this pregnancy (before 20 weeks’ gestation)? *(Multiple choice)*
2. Yes
3. No (skip to Q19)
4. Don't remember (skip to Q19)
5. Never smoked (skip to Q19)
6. How many cigarettes did you smoke in a typical day at the beginning of this pregnancy (before 20 weeks' gestation)? *(Multiple choice)*
7. 1-10
8. 11-20
9. 21-30
10. 31-40
11. More than 40
12. Were you provided with information about the risks associated with smoking and the benefits of quitting at your first antenatal booking visit? *(Multiple choice)*
13. Yes
14. No
15. Don't remember
16. Were you referred to a stop smoking service? *(Multiple choice)*
17. Yes
18. No (skip to Q13)

- Is there anything you would like to tell us about this referral process? (Open text, optional question)
- Please tell us about this: *(Open text, optional question)*

1. Did you use this service? *(Multiple choice)*
2. Yes
3. No

- Please tell us which service and anything you might like to share about your experience at this service: *(Open text, optional question)*
- Please provide any comments about why you did not attend a stop smoking service: *(Open text, optional question)*

1. Thinking about the support you received during your pregnancy to help you stop smoking: (Matrix)

|  | Very unsatisfied | Unsatisfied | Neutral | Satisfied | Very satisfied |
| --- | --- | --- | --- | --- | --- |
| How satisfied are you with the support you received from the healthcare provider/hospital you attended? |  |  |  |  |  |
| How satisfied are you with the support you received from the stop smoking service you were referred to? |  |  |  |  |  |

1. Were you able to quit smoking during your pregnancy? *(Multiple choice)*
2. Yes
3. No
4. If you were able to stop smoking during this pregnancy, can you please tell us what stage of pregnancy this was (in trimesters) (Multiple choice)
   1. First trimester (0-12 weeks)
   2. Second trimester (13-26 weeks)
   3. Third trimester (27-40 weeks)
5. If you were able to cut down the number of cigarettes you typically smoked in a day during this pregnancy, can you please tell us what stage of pregnancy this was (in weeks). *(Multiple choice)*
   1. First trimester (0 to 12 weeks)
   2. Second trimester (13 to 26 weeks)
   3. Third trimester (27 to 40 weeks) I wasn't able to cut down
6. Were you smoking around the time you had your baby? (Multiple choice)
   1. Yes
   2. No (skip to Q19)
   3. Don't remember (skip to Q19)
7. How many cigarettes did you smoke in a typical day around the time you had your baby? *(Multiple choice)*
   1. 1-10
   2. 11-20
   3. 21-30
   4. 31-40
   5. More than 40
8. At your booking appointment (usually your first appointment at the hospital or booking history over the phone) were you asked by your midwife/doctor whether you smoked cigarettes (multiple choice)
   1. Yes
   2. No
   3. I reported I did not smoke before I was asked
   4. Don’t remember
9. ***POST IMPLEMENTATION SURVEY QUESTION ONLY*** At your booking appointment (usually your first appointment at the hospital or booking history over the phone) were you asked by your midwife/doctor whether you are regularly exposed to passive cigarette smoke (breathing in other people’s cigarette smoke)
   1. Yes
   2. No
   3. Don’t remember
10. Were you offered a breath test (with a monitor like the one pictured) to measure the level of carbon monoxide? *(Multiple choice)*
    1. Yes
    2. No (skip to Q23)
    3. Don't remember (skip to Q23)
    4. Not applicable (skip to Q23)
11. When were you offered a breath test? *(Multiple choice)*
    1. At my first antenatal booking visit only
    2. Later in pregnancy at around my 28 weeks antenatal visit only
    3. At my first antenatal booking visit and later in pregnancy at around my 28 weeks antenatal visit
    4. I declined to take the test
    5. Other:

- Is there anything would like to tell us about how you felt when your midwife/doctor talked to you about stopping smoking during pregnancy or breath testing? (Open text, optional question)

**Section 4: Element 2 (Fetal Growth Restriction)**

1. During your pregnancy did the midwife or doctor use a tape measure on your tummy to measure the growth of your baby? *(Multiple choice)*
   1. Yes, at every antenatal appointment (from about 28 weeks' gestation)
   2. Yes, but only at one or some antenatal appointments (from about 28 week's gestation)
   3. No
   4. Don’t remember
2. During your pregnancy did you have ultrasound scans in the third trimester (last 3 months) of your pregnancy? *(Multiple choice)*
   1. Yes, at every antenatal appointment
   2. Yes, from external provider (separate to usual antenatal appointments) Yes, but only at one or some antenatal appointments
   3. No
   4. Don't remember
3. Thinking about the information you received during your pregnancy about how your baby was growing: *(Multiple choice)*

|  | Very unsatisfied | Unsatisfied | Neutral | Satisfied | Very satisfied |
| --- | --- | --- | --- | --- | --- |
| How satisfied are you with the information you received from the healthcare provider/ hospital you attended? |  |  |  |  |  |

- Is there anything you would like to tell us about how you felt when your midwife/doctor talked to you about the growth of your baby in the third trimester (last 3 months) of your pregnancy? (Open text, optional question)

**Section 5: Decreased Fetal Movements**

1. What happens to babies' movements towards the end of pregnancy? *(Multiple choice)*
   1. Movements stop
   2. Babies move less because they're running out of room
   3. Babies move more
   4. Babies move about the same amount
   5. Don't know
2. What should you do if you feel your baby is moving less than usual? *(Multiple choice)*
   1. Lie on your side for two hours and see if you can count 10 movements
   2. Contact your midwife/doctor immediately
   3. Double check your baby is okay with a home monitor
   4. Wait until the next day to see if things improve
   5. Have a cold drink or something to eat and try to make the baby move
   6. Don't know
3. Other than asking you if you had felt your baby move, did your midwife/doctor discuss your baby’s movements with you and what to do if you were concerned about movements at all during your pregnancy? *(Multiple choice)*
   1. Yes, at every antenatal appointment (from about 28 weeks)
   2. Yes, but only at one or some antenatal appointments (from about 28 weeks)
   3. No
   4. Don’t remember
4. Were you concerned at any point during this pregnancy that your baby’s movements had changed? *(Multiple choice)*
   1. Yes
   2. No (skip to Q36)
   3. Don't remember (skip to Q36)
5. Did you call your healthcare provider/hospital when you were concerned about your baby’s movements? *(Multiple choice)*
   1. Yes
   2. Yes, but I was unable to speak to them (skip to Q32)
   3. No (skip to Q32)
6. When you were concerned about your baby’s movements, how soon did you contact your healthcare provider/hospital (hours)? *(Multiple choice)*
   1. 0 to 2 hours
   2. 3 to 11 hours
   3. 12 to 24 hour
   4. 24 to 48 hours
   5. More than 48 hours
7. Did you visit your healthcare provider/hospital when you were concerned about your baby’s movements? *(Multiple choice)*
   1. Yes
   2. No (skip to Q36)
8. Approximately how many weeks pregnant were you? *(Open text)*
9. How many times did you visit your healthcare provider/hospital when this happened? *(Open text)*

- Please tell us about this: *(Open text, optional question)*

1. If you did visit your healthcare provider/hospital maternity unit when you were concerned about your baby’s movements, please indicate when (after how many hours)? *(Multiple choice)*
   1. 0-2 hours
   2. 3-12 hours
   3. 12-24 hours
   4. 24-48 hours
   5. More than 48 hours

- Please tell us why: (Open text, optional question)

1. Thinking about the care you received in the third trimester (last 3 months) about your baby’s movements: *(Multiple choice)*

|  | Very unsatisfied | Unsatisfied | Neutral | Satisfied | Very satisfied |
| --- | --- | --- | --- | --- | --- |
| How satisfied are you with the information you received about your baby’s movements from the healthcare provider/hospital you attended? |  |  |  |  |  |
| If at any time during your pregnancy you were concerned about your baby’s movements, how satisfied were you with the care you received from the healthcare provider/hospital you attended? |  |  |  |  |  |

- Is there anything you would like to tell us about how you felt when your midwife/doctor talked to you about your baby’s movements during pregnancy? (Open text, optional question)

**Section 6: Going-to-sleep position in the third trimester of pregnancy**

1. For all episodes of sleep, please indicate what a safe going‐to‐sleep position is in late pregnancy (after 28 weeks’ gestation): *(Tick all that apply)*
   1. Back Tummy
   2. Left side
   3. Right side
   4. Sitting propped up
   5. Don't know
2. What was your usual going‐to‐sleep position in late pregnancy? *(Multiple choice)*
   1. Back
   2. Tummy
   3. Left side
   4. Right side
   5. Both left and right side
   6. Sitting propped up
   7. Position varied (back, side, tummy, propped up)
   8. Don't remember
3. Did you try to avoid going‐to‐sleep in any particular position in late pregnancy? *(Tick all that apply)*
   1. Yes, on my back
   2. Yes, on my tummy
   3. Yes, on my left side
   4. Yes, on my right side
   5. Yes, propped up
   6. No
   7. Don't remember
4. Did you change your going‐to‐sleep position in late pregnancy because of the advice or information you received? *(Multiple choice)*
   1. Yes
   2. No
5. Did your midwife/doctor discuss with you the importance of sleeping on your side in late pregnancy? *(Multiple choice)*
   1. Yes, at every antenatal appointment (from about 28 weeks)
   2. Yes, but only at one or some antenatal appointments (from about 28 weeks)
   3. No
   4. Don't remember
6. Thinking about the information you received during your pregnancy about going‐to‐sleep positions in the third trimester (last 3 months) of pregnancy: *(Multiple choice)*

|  | Very unsatisfied | Unsatisfied | Neutral | Satisfied | Very satisfied |
| --- | --- | --- | --- | --- | --- |
| How satisfied are you with the information you received about going‐to‐sleep positions in late pregnancy from the healthcare provider/ hospital you attended? |  |  |  |  |  |

- Is there anything you would like to tell us about how you felt when your midwife/doctor talked to you about your going‐to‐sleep position in the third trimester (last 3 months) of your pregnancy? (Open text, optional question)

**Section 7: Timing of Birth**

1. At your antenatal booking appointment did your midwife/doctor talk with you about your risk of having a stillborn baby? *(Multiple choice)*
   1. Yes
   2. No
   3. Unsure (as I'm not sure what the risk factors are)
   4. Don't remember

- If you had any risk factors, what were they? *(Open text, optional question)*

1. Throughout your pregnancy, did your midwife/doctor talk with you about your risk of having a stillborn baby? *(Multiple choice)*
   1. Yes, at every antenatal appointments early in my pregnancy
   2. Yes, at antenatal appointments late in my pregnancy (around 34-36 weeks)
   3. Yes, at antenatal appointments both early and late in my pregnancy
   4. No
   5. Don't remember

- If you had any risk factors, what were they? *(Open text, optional question)*
- Is there anything you would like to tell us about how you felt when your midwife/doctor talked to you about your risk factors for stillbirth? *(Open text, optional question)*

1. Thinking about the information you received during your pregnancy about the risk factors for having a stillborn baby: *(Multiple choice)*

|  | Very unsatisfied | Unsatisfied | Neutral | Satisfied | Very satisfied |
| --- | --- | --- | --- | --- | --- |
| How satisfied are you with the information you received from the healthcare provider/ hospital you attended? |  |  |  |  |  |

1. Did your midwife/doctor talk with you about the possibility of you having a planned birth (ie: scheduled date and time for birth rather than waiting for labour to start on its own)?
   1. Yes, at antenatal appointments early in my pregnancy
   2. Yes, at antenatal appointments late in my pregnancy (around 34-36 weeks)
   3. Yes, at antenatal appointments both early and late in my pregnancy
   4. No (skip to Q48)
   5. Don't remember (skip to Q48
2. At what gestation (weeks) did your midwife/doctor recommend that the birth occur? *(Open text)*
3. Thinking about your experiences when making decisions and choosing options about the timing of your baby's birth this pregnancy: *(Multiple choice)*

|  | Strongly disagree | Disagree | Neutral | Agree | Strongly agree |
| --- | --- | --- | --- | --- | --- |
| My midwife or doctor asked me how involved in decision‐making I wanted to be |  |  |  |  |  |
| My midwife or doctor told me that there are different options for my maternity care |  |  |  |  |  |
| My midwife or doctor explained the advantages and disadvantages of the maternity care options |  |  |  |  |  |
| My midwife or doctor helped me understand all the information |  |  |  |  |  |
| I was given enough time to thoroughly consider the different maternity options |  |  |  |  |  |
| I was able to choose what I considered to be the best options |  |  |  |  |  |
| My doctor or midwife respected my choice |  |  |  |  |  |
| My midwife or doctor discussed any worries or fears that I had about the timing of my baby's birth |  |  |  |  |  |

1. Were you involved as much as you wanted to be when making decisions and choosing options about the timing of your baby's birth? *(Multiple choice)*
   1. Yes
   2. No
   3. Don't remember

- Is there anything else you would like to tell us about the care and information you received during pregnancy about your risk for stillbirth or planning the timing of your baby’s birth? (Open text, optional question)

**Section 8: Demographics**

1. Is Australia your country of birth? (Multiple choice)
   1. Yes
   2. No
2. What is your country of birth? *(Open text)*
3. Are you of Aboriginal or Torres Strait Islander origin? *(Multiple choice)*
   1. Yes, Aboriginal (skip to Q54)
   2. Yes, Torres Strait Islander (skip to Q54)
   3. Yes, both Aboriginal and Torres Strait Islander (skip to Q54)
   4. No
4. What is your ethnicity? *(Multiple choice)*
   1. Maori/ Pacific Islander
   2. Papua New Guinean, Timorese
   3. Caucasian
   4. South Asian (Indian, Pakistani, Bangladeshi, Sri Lankan)
   5. Mainland Southeast Asian (Cambodian, Lao, Viet, Thai)
   6. Malay, Philippino, Indonesian
   7. Chinese
   8. Japanese
   9. Middle Eastern and North African
   10. Sub-Saharan African
   11. Central and South America
   12. Other:
5. Is your first language English? *(Multiple choice)*
   1. Yes (skip to Q56)
   2. No
6. Please specify your first language: *(Open text)*
7. Age (years) at time of giving birth? *(Multiple choice)*
   1. 18 to 24 years
   2. 25 to 34 years
   3. 35 to 44 years
   4. 45 to 54 years
   5. 55 years or more
   6. Prefer not to say
8. How many people do you have near you that you can readily count on for help in times of difficulty such as to watch over children or pets, give rides to the hospital or store, or help when you are sick? (Multiple choice)
   1. 0
   2. 1
   3. 2 - 5
   4. 6 - 10
   5. More than 10
9. What is your highest level of education? (Multiple choice)
   1. None
   2. Primary school
   3. High school
   4. TAFE certificate or diploma
   5. Undergraduate university degree
   6. Post graduate university degree
   7. Doctorate
10. Is your baby of Aboriginal or Torres Strait Islander origin? *(Multiple choice)*
    1. Yes, Aboriginal
    2. Yes, Torres Strait Islander
    3. Yes, both Aboriginal and Torres Strait Islander
    4. No
11. At how many weeks gestation was your baby born? *(Open text)*
12. Was your baby admitted to neonatal nursery/special care unit? *(Multiple choice)*
    1. Yes
    2. No
13. Was the timing of your baby’s birth planned (ie. scheduled date and time for birth rather than waiting for labour to start on its own)? (Multiple choice)
    1. No, I went into labour naturally without intervention (spontaneous labour and birth) (skip to Q64)
    2. Yes, I had a planned induction of labour
    3. Yes, I had a planned caesarean section
14. At what gestation (weeks) was your induction/caesarean planned for? *(Open text)*
15. How did you give birth to your baby? *(Multiple choice)*
    1. Unassisted vaginal birth
    2. Ventouse or vacuum (suction cup)
    3. Forceps
    4. Emergency caesarean section
    5. Elective (planned) caesarean section
16. Prior to your most recent pregnancy, have you been pregnant before? *(Multiple choice)*
    1. Yes
    2. No (skip to Q67)
17. Did you experience any of the following outcomes in your previous pregnancy/ies? *(Tick all that apply)*
    1. Miscarriage (pregnancy loss before 20 weeks)
    2. Stillbirth (pregnancy loss after 20 weeks (5 months)
    3. Early newborn death (baby died within 28 days of birth)
    4. Not applicable, I did not experience any of these
    5. Other:
18. In which state or territory was the hospital in which you received most of your antenatal care? *(Drop down list)*
19. Through which maternity service did you receive the most of your antenatal care? *(Drop down list of Queensland and New South Wales Hospitals)*
20. Through which maternity service did you give birth? *(Drop down list of Queensland and New South Wales Hospitals)*
