# Supplementary material for: Experiences of antenatal care practices to reduce stillbirth: surveys of women and healthcare professionals pre-post implementation of the Safer Baby Bundle
Source: BMC Pregnancy Childbirth. 2024 Aug 1;24:520. doi: 10.1186/s12884-024-06712-8 (PMC11295589; doi:10.1186/s12884-024-06712-8)
Supplement: Supplementary file 2 — Supplementary Material 2 [file 12884_2024_6712_MOESM2_ESM.docx]

**Additional file 2: Healthcare professional’s online survey tool**

**Survey of healthcare professionals in Australia** **– Post implementation of the Safer Baby Bundle**

**Section 1: Demographics**

1. In which state or territory is the maternity service you currently work? (Drop down list)
2. In which maternity service do you currently work? (Drop down list of Queensland and New South Wales Hospitals)
3. Which best describes your maternity service? (Multiple choice)
   1. Public Hospital Only
   2. Private Hospital Only
   3. Both Public & Private
   4. Other:
4. What is your discipline? (Multiple choice)
5. Student/Pre-registration healthcare worker Midwife
6. Nurse Doctor
7. General Practitioner (GP) Medical Officer
8. Resident Medical Officer Obstetrician
9. Obstetric Registrar Gynaecologist Neonatologist
10. Aboriginal and Torres Strait Islander Health Worker Allied Health Worker
11. Sonographer or radiologist Psychologist
12. Other:
13. What is your primary work area(s) (clinical area or department in which you spend most of your work time/provide most of your clinical services) in the maternity unit? (Tick all that apply)
14. Midwifery group practice providing continuity of care Pregnancy assessment unit
15. Antenatal inpatient Specialty antenatal clinic Birthing unit
16. Postnatal in patient care or home care Private midwifery practice
17. Antenatal education Education
18. Research
19. Ultrasound department
20. Maternal fetal medicine department GP shared care
21. Other
22. Please select your years of experience (Multiple choice)
23. Student in training
24. Less than 1 years' experience
25. Within 2-5 years’ experience
26. Within 5-10 years’ experience
27. More than 10 years’ experience
28. What is your gender? (Multiple choice)
29. Male
30. Female
31. Prefer not to say
32. Other

**Section 2: Awareness of the Safer Baby Bundle.**

1. ***POST IMPLEMENTATION SURVEY QUESTION ONLY*** Prior to participating in this survey, had you heard about the Safer Baby Bundle (Multiple choice)
2. Yes
3. No (skip to Q11)
4. ***POST IMPLEMENTATION SURVEY QUESTION ONLY*** Were you aware that the Safer Baby Bundle has been implemented at your service? (Multiple choice)
5. Yes (skip to Q11)
6. No
7. Unsure

- *Can you please tell us how you heard about the Safer Baby Bundle: (Open text)*

1. ***POST IMPLEMENTATION SURVEY QUESTION ONLY*** How were you made aware of the Safer Baby Bundle implementation at your service? (Tick all that apply)
2. Attended a launch day (In-person or virtual)
3. Study day held at the hospital (Face-to-face workshop)
4. Incorporated into in-service education
5. Peer-to-peer communication
6. eLearning and webinars
7. Unsure
8. Other:

- Is there anything more you would like to tell us about how you were made aware of the Safer Baby Bundle? (Open text, optional question)

**Section 2: supporting women to stop smoking during pregnancy**

1. In your current role, do you provide antenatal care to women where you can support quitting smoking during pregnancy? (Multiple choice)
2. Yes
3. No (skip to Q17)
4. Unsure
5. Please indicate the frequency with which you perform the following: (Multiple choice)

|  | All the time | Most of the time | Half of the time | Not much of the time | Never | Not applicable |
| --- | --- | --- | --- | --- | --- | --- |
| Record/document a woman’s smoking status at the first antenatal care visit (booking in visit) |  |  |  |  |  |  |
| For women who are smokers or recent quitters, provide information and advice on the benefits of quitting |  |  |  |  |  |  |
| Offer women who smoke personalised advice on how to stop smoking (e.g. setting quit date, making quit plan) |  |  |  |  |  |  |
| Refer woman to Quitline (or other locally appropriate smoking cessation service) if identified as a smoker |  |  |  |  |  |  |
| Record passive smoking status at the first antenatal care visit |  |  |  |  |  |  |
| Refer a woman’s partner to Quitline (or other locally appropriate smoking cessation service) if they smoke |  |  |  |  |  |  |
| Ask women if they attended their referral appointment to Quitline (or other locally appropriate smoking cessation service) |  |  |  |  |  |  |
| For women identified as smokers or recent quitters, use the Ask, Advise and Help brief advice model to follow‐up at every antenatal visit |  |  |  |  |  |  |
| Offer all women (regardless of smoking status) an exhaled breath carbon monoxide (CO) reading (and their partners where available) |  |  |  |  |  |  |

- *Please specify why you indicated not applicable (Open text, optional question)*

1. How do you assess a woman’s smoking status at the first antenatal care visit (tick all that apply)? (Tick all that apply)
2. Verbally (ask)
3. Verbally using a multi-choice format (e.g. which of these best describes your experience? I smoke more since pregnant/ I smoke less since pregnant/ I am smoking the same/ I used to smoke but quit/ I have never smoked)
4. The woman completes a self-report form
5. An exhaled breath carbon monoxide test
6. Other:
7. Do you provide women with access to the SBB Quit smoking for baby brochure at an antenatal visit? (Drop down list)
8. Yes
9. No, I provide a different brochure on smoking cessation
10. No, I do not provide any brochure on smoking cessation
11. I don't know about this brochure

- *Please specify which other brochure you provide (Open text)*
- *Please specify why you do not provide a brochure (Open text)*

1. How satisfied are you with the support your maternity service provides to women during pregnancy to help them to stop smoking? (Multiple choice)
2. Very unsatisfied
3. Unsatisfied
4. Neither satisfied nor dissatisfied
5. Satisfied
6. Very satisfied
7. Not applicable
8. Is there anything else you would like to tell us about the current information/resources you provide women? Are there any ways in which these could be improved? (Open text, optional question)
9. Thinking about having a conversation with women about smoking cessation support during antenatal visits, please answer the following: (Multiple choice)

|  | Strongly disagree | Disagree | Neither agree nor disagree | Agree | Strongly agree | Not applicable |
| --- | --- | --- | --- | --- | --- | --- |
| I have adequate time to provide appropriate care |  |  |  |  |  |  |
| I feel confident about my level of knowledge |  |  |  |  |  |  |
| I feel comfortable discussing this with women |  |  |  |  |  |  |
| I have concerns that this conversation will cause anxiety for women |  |  |  |  |  |  |
| I have concerns this conversation will negatively impact on my relationship with the woman |  |  |  |  |  |  |

- *Is there anything else you would like to tell us about how you feel having these conversations with women? (Open text, optional question)*

**Section 3: Fetal Growth Restriction (FGR) risk assessment and management**

1. In your current role, do you provide antenatal care to women related to assessing and monitoring their baby's growth? (Multiple choice)
2. Yes
3. No (skip to Q23)
4. Unsure
5. Please indicate the frequency with which you perform the following: (Multiple choice)

|  | All the time | Most of the time | Half of the time | Not much of the time | Never | Not applicable |
| --- | --- | --- | --- | --- | --- | --- |
| Early in pregnancy, do you assess all women with a singleton pregnancy for risk factors for FGR? |  |  |  |  |  |  |
| At every subsequent antenatal visit from 24 weeks’ gestation do you assess women for risk factors for FGR |  |  |  |  |  |  |
| At antenatal visits after 24 weeks’ gestation, for all women, do you take SFH measurements (unless unsuitable due to maternal factors)? |  |  |  |  |  |  |
| At antenatal visits after 24 weeks’ gestation, for all women, do you plot SFH measurements on a growth chart (unless not suitable due to maternal factors)? |  |  |  |  |  |  |
| Refer women at increased risk of FGR (level 3) for growth scans every 2‐3 weeks from 24 weeks gestation until birth. |  |  |  |  |  |  |

- *Please specify why you indicated not applicable for any of the above responses (Open text, optional question)*

1. Do you use the PSANZ/Stillbirth CRE Fetal Growth Restriction (FGR) Care Pathway (FGR care Pathway) to classify a woman’s risk of a fetal growth restriction? (Multiple choice)
2. Yes (Skip to Q22)
3. No, I use a different care pathway/algorithm
4. No, I don’t use any care pathway/algorithm (Skip to Q22)
5. Don’t know (Skip to Q22)
6. Please specify which care pathway/algorithm you use: (multiple choice)
7. NSW Fetal Safety Risk Assessment Pathway: Fetal Growth Restriction
8. Other (open text)
9. Do you provide women with access to the SBB Fetal Growth Restriction brochure before 28 weeks’ gestation? (Multiple choice)
10. Yes
11. No, I provide a different brochure on FGR
12. No, I do not provide any brochure on FGR
13. I don't know about this brochure
14. Not applicable

- *Please specify which brochure you provide (Open text, optional question)*
- *Please specify why you do not provide a brochure (Open text, optional question)*

1. How satisfied are you with the information your maternity service provides to women during pregnancy about monitoring their baby's growth? (Multiple choice)
2. Very unsatisfied
3. Unsatisfied
4. Neither satisfied nor dissatisfied
5. Satisfied
6. Very satisfied
7. Not applicable

- *Is there anything you would like to tell us about the current information/resources you provide to women? Are there any ways in which these could be improved? (Open text, optional question)*

1. Thinking about having a conversation with women about their risk factors for having a small‐for‐ gestational‐age fetus/newborn during antenatal visits, please answer the following: (Multiple choice)

|  | Strongly disagree | Disagree | Neither agree nor disagree | Agree | Strongly agree | Not applicable |
| --- | --- | --- | --- | --- | --- | --- |
| I have adequate time to provide all the information |  |  |  |  |  |  |
| I have good knowledge of the evidence‐based information to provide to women |  |  |  |  |  |  |
| I feel comfortable discussing this with women |  |  |  |  |  |  |
| I have concerns that this conversation will cause anxiety for women |  |  |  |  |  |  |
| I have concerns that this conversation will negatively impact on my relationship with them |  |  |  |  |  |  |

- *Is there anything else you would like to tell us about how you feel about having these conversations with women? (Open text, optional question)*

**Section 4: awareness and management of women with decreased fetal movements**

1. In your current role, do you provide care to women regarding the awareness and management of decreased/altered fetal movements? (Multiple choice)
2. Yes
3. No (Skip to Q30)
4. Don't know
5. How do you provide information to a woman relating to fetal movements? (Tick all that apply)
6. Verbally
7. Written information
8. Websites
9. Mobile phone (e.g. smartphone app or SMS-based program)
10. Not applicable
11. Other:

- *Please specify (Open text, optional question*)

1. Please indicate the frequency with which you perform the following: (Multiple choice)

|  | All the time | Most of the time | Half of the time | Not much of the time | Never | Not applicable |
| --- | --- | --- | --- | --- | --- | --- |
| At each antenatal visit after 28 weeks’ gestation, I remind women of the importance of reporting DFM and to report concerns without delay |  |  |  |  |  |  |
| For women from 28 weeks’ gestation who present/ attend your service with concerns about DFM, how often is a CTG commenced within 2 hours of presentation |  |  |  |  |  |  |

- *Please specify why you indicated not applicable (Open text, optional question)*

1. If a woman presents with concerns about DFM (from 28 weeks’ gestation), would you refer her for an ultrasound if: (1) normal CTG and clinical assessment; and (2) no risk factors identified for stillbirth; and (3) first presentation for DFM; and (4) no maternal concerns of DFM at time of assessment? (Multiple choice)
2. Yes, definitely
3. No I wouldn’t
4. Sometimes

- *Please specify (Open text, optional question)*

1. Do you provide women with access to the Movements Matter brochure on DFM before 28 weeks’ gestation? (Drop down list)
2. Yes
3. No, I provide a different brochure on DFM
4. No, I don't provide a brochure on DFM
5. I don't know about this brochure
6. Not applicable

- *Please specify which brochure you provide (Open text, optional question)*
- *Please specify why you do not provide a brochure (Open text, optional question)*

1. How satisfied are you with the support your maternity service provides to women during pregnancy about monitoring their baby’s movements? (Multiple choice)
2. Very unsatisfied
3. Unsatisfied
4. Neither satisfied nor dissatisfied
5. Satisfied
6. Very satisfied
7. Not applicable

- *Is there anything else you would like to tell us about the current information/resources you provide women? Are there way in which these could be improved? (Open text, optional question)*

1. Thinking about having a conversation with women who present with concerns about fetal movements, please answer the following: (Multiple choice)

|  | Strongly disagree | Disagree | Neither agree nor disagree | Agree | Strongly agree | Not applicable |
| --- | --- | --- | --- | --- | --- | --- |
| I have adequate time to provide appropriate care  I have adequate time to provide appropriate care |  |  |  |  |  |  |
| I feel confident about my level of knowledge |  |  |  |  |  |  |
| I feel comfortable discussing this with women |  |  |  |  |  |  |
| I have concerns that this conversation will cause anxiety for women |  |  |  |  |  |  |
| I have concerns that this conversation will negatively impact on my relationship with the woman |  |  |  |  |  |  |

- Is there anything else you would like to tell us about how you feel about having these conversations with women? (Open text, optional question)

**Section 5: Maternal safe going‐to‐sleep position in late pregnancy**

1. Please indicate the frequency with which you perform the following: (Multiple choice)

|  | All the time | Most of the time | Half of the time | Not much of the time | Never | Not applicable |
| --- | --- | --- | --- | --- | --- | --- |
| Provide written information and discuss maternal safe going‐to‐sleep position in late pregnancy with women by, at the latest, week 28 of pregnancy |  |  |  |  |  |  |
| Discuss maternal safe going-to-sleep position in late pregnancy with pregnant women at every antenatal visit after 28 weeks’ gestation |  |  |  |  |  |  |

- *Please specify why you indicated not applicable (open text, optional question)*

1. Do you provide women with access to the Sleep on your side brochure before 28 weeks’ gestation?

(Multiple choice)

1. Yes
2. No, I do not provide any brochure on side sleep
3. No, I provide a different brochure
4. I don't know about this brochure
5. Not applicable

- *Please specify which brochure you provide* (Open text, optional question)
- *Please specify why you do not provide a brochure* (Open text, optional question)

1. How satisfied are you with the information your maternity service provides to women during the third trimester of pregnancy about maternal safe going‐to‐sleep position in late pregnancy? (Multiple choice)
2. Very unsatisfied
3. Unsatisfied
4. Neither satisfied nor dissatisfied
5. Satisfied
6. Very satisfied
7. Not applicable

- *Is there anything else you would like to tell us about the current information/resources you provide to women? Are there any ways in which these could be improved? (Open text, optional question)*

1. Thinking about having a conversation with women about maternal safe going‐to‐sleep position in late pregnancy, please answer the following: (Multiple choice)

|  | Strongly disagree | Disagree | Neither agree nor disagree | Agree | Strongly agree | Not applicable |
| --- | --- | --- | --- | --- | --- | --- |
| I have adequate time to provide appropriate care |  |  |  |  |  |  |
| I feel confident about my level of knowledge |  |  |  |  |  |  |
| I feel comfortable discussing this with women |  |  |  |  |  |  |
| I have concerns that this conversation will cause anxiety for women |  |  |  |  |  |  |
| I have concerns that this conversation will negatively impact on my relationship with the woman |  |  |  |  |  |  |

- *Is there anything else you would like to tell us about how you feel about having these conversations with women? (Open text, optional question)*

**Section 6: Assessing the risks and benefits around timing of birth for women with risk factors for stillbirth**

1. As part of your antenatal care practice, do you have a conversation with women about their risk of having a stillborn baby (explicitly using the word stillbirth)? (Multiple choice)
2. Yes
3. No (Skip to Q17)
4. Not applicable (Skip to Q)
5. At approximately what gestation (trimester) do you first have this conversation? (Multiple choice)
6. First trimester (0-12 weeks)
7. Second trimester (13-26 weeks)
8. Third trimester (27-40 weeks)
9. Other:
10. Do you have this conversation with all women regardless of their risk status for having a stillborn baby? (Multiple choice)
11. Yes
12. No
13. Not applicable

- *Please specify why you indicated no or not applicable (Open text)*

1. Please indicate the frequency with which you perform the following: (Multiple choice)

|  | All the time | Most of the time | Half of the time | Not much of the time | Never | Not applicable |
| --- | --- | --- | --- | --- | --- | --- |
| How often do you assess all women for stillbirth risk factors at the first antenatal care visit and document on the woman’s notes? |  |  |  |  |  |  |
| Reassess all women for stillbirth risk at 34‐36+6 weeks’ gestation and document on the woman’s notes |  |  |  |  |  |  |
| How often do you discuss birth planning according to a women’s risk status for stillbirth? |  |  |  |  |  |  |
| Provide women with individualised information based on risk assessment to support informed, shared decision‐ making on timing of birth |  |  |  |  |  |  |

- *Please clarify why you indicated "Not applicable": (Open text, optional question)*

1. How satisfied are you with the information your maternity service provides to women on risks and benefits around timing of birth based on the woman’s individual profile of risk factors? (Multiple choice)
2. Very unsatisfied
3. Unsatisfied
4. Neither satisfied nor dissatisfied
5. Satisfied
6. Very satisfied
7. Not applicable

- *Is there anything else you would like to tell us about the current information/resources on risks and benefits around timing of birth that you provide to women? Are there any ways in which these could be improved? (Open text, optional question)*

1. Thinking about having a conversation with women about the risks and benefits around timing of birth based on the woman’s individual profile of stillbirth risk, please answer the following: (Multiple choice)

|  | Strongly disagree | Disagree | Neither agree nor disagree | Agree | Strongly agree | Not applicable |
| --- | --- | --- | --- | --- | --- | --- |
| I have adequate time to provide appropriate care |  |  |  |  |  |  |
| I feel confident about my level of knowledge |  |  |  |  |  |  |
| I feel comfortable discussing this with women |  |  |  |  |  |  |
| I have concerns that this conversation will cause anxiety for women |  |  |  |  |  |  |
| I have concerns this conversation will negatively impact on my relationship with the woman |  |  |  |  |  |  |
| I feel comfortable with involving women as much as they want to be in the decision‐making process about timing of birth |  |  |  |  |  |  |

- *Is there anything else you would like to tell us about how you feel about having these conversations with women? (Open text)*
- *Are there any factors or resources you feel would better enable you, in your everyday practice, to have conversations with women about their risk of stillbirth? (Open text)*

**Section 7: Impression of the SBB initiative and resources.**

1. ***POST IMPLEMENTATION SURVEY QUESTION ONLY*** Do you feel adequately trained in how to best support women in regard to the following Safer Baby Bundle elements of care? (Multiple choice)

|  | Yes, no need for more training | Yes, but I would like more training | No | Not applicable |
| --- | --- | --- | --- | --- |
| Supporting women to stop smoking during pregnancy (e.g. CO monitor use and/or using the ask, advise and help brief advice model). |  |  |  |  |
| Detection and management of fetal growth restriction (e.g. SFH measurement and plotting on growth charts). |  |  |  |  |
| Improving care for women with decreased fetal movements. |  |  |  |  |
| Maternal safe going‐to‐sleep position in late pregnancy (e.g. having conversations with women around maternal safe going‐to‐sleep positions). |  |  |  |  |
| Decision‐making about the timing of birth for women with risk factors for stillbirth (e.g. communication with women about stillbirth risk and optimal timing of birth). |  |  |  |  |

1. ***POST IMPLEMENTATION SURVEY QUESTION ONLY*** What approaches does your maternity service use to provide training in the SBB elements of care? (Tick all that apply)
2. Attended a launch day (in-person or virtual)
3. Study day held at the hospital (face-to-face workshop) )
4. Incorporated into in service education
5. Peer-to-peer communication eLearning and webinars
6. Unsure
7. Other:

- *Are there any specific approaches that your maternity service has used to provide training in the SBB elements of care that you would like to tell us about? (Open text)*

1. ***POST IMPLEMENTATION SURVEY QUESTION ONLY*** Does your service provide women with access to Safer Baby Bundle resources? (Multiple choice)
2. Yes
3. No
4. Not sure
5. Not applicable
6. ***POST IMPLEMENTATION SURVEY QUESTION ONLY*** Considering the Safer Baby Bundle initiative and related resources, please answer the following questions: (Multiple choice)

|  | Strongly disagree | Disagree | Neither agree nor disagree | Agree | Strongly agree |
| --- | --- | --- | --- | --- | --- |
| The Safer Baby Bundle has improved the quality of antenatal care I provide. |  |  |  |  |  |
| The Safer Baby Bundle has improved the quality of antenatal care provided by the service in which I work. |  |  |  |  |  |
| The Safer Baby Bundle is effective |  |  |  |  |  |
| The Safer Baby Bundle recommendations are evidence‐based. |  |  |  |  |  |
| I regularly use the Safer Baby Bundle to inform my practice. |  |  |  |  |  |
| I have enough time in my everyday practice to follow the Safer Baby Bundle recommendations  I feel like the recommendations of the Safer Baby Bundle have become part of my routine practice. |  |  |  |  |  |
| The Safer Baby Bundle elements of care have been well implemented at my maternity service. |  |  |  |  |  |

1. ***POST IMPLEMENTATION SURVEY QUESTION ONLY*** What is your impression of the impact of implementing the SBB elements at your service? (Multiple choice)

|  | Extremely negative | Somewhat negative | Neutral | Positive | Extremely positive |
| --- | --- | --- | --- | --- | --- |
| Overall |  |  |  |  |  |
| Smoking cessation (Element 1) |  |  |  |  |  |
| Fetal Growth Restriction (Element 2) |  |  |  |  |  |
| Decreased Fetal Movements (Element 3) |  |  |  |  |  |
| Sleeping on your side (Element 4) |  |  |  |  |  |
| Timing of Birth (Element 5) |  |  |  |  |  |
