# Supplementary material for: Experiences of antenatal care practices to reduce stillbirth: surveys of women and healthcare professionals pre-post implementation of the Safer Baby Bundle
Source: BMC Pregnancy Childbirth. 2024 Aug 1;24:520. doi: 10.1186/s12884-024-06712-8 (PMC11295589; doi:10.1186/s12884-024-06712-8)
Supplement: Supplementary file 3 — Supplementary Material 3 [file 12884_2024_6712_MOESM3_ESM.docx]

**Additional file 3.**

Provision of best practice recommendations and information related to stillbirth risk and SBB elements.

| **Recommendation** | **Source** | **Response** | **Pre n (%)** | **Post n (%)** |
| --- | --- | --- | --- | --- |
| Record smoking status at first antenatal visit | HCP | All the time | 836 (85.9%) | 200 (88.9%) |
|  |  | Most of the time | 67 (6.9%) | 16 (7.1%) |
|  |  | Half of the time | 10 (1.0%) | 4 (1.8%) |
|  |  | Not much of the time | 35 (3.6%) | 5 (2.2%) |
|  |  | Never | 25 (2.6%) | 0 (0.0%) |
| Asked at booking appointment whether you smoked | Women | Yes | 985 (89.9%) | 112 (86.8%) |
|  |  | No | 44 (4.0%) | 7 (5.4%) |
|  |  | I reported I did or did not smoke before I was asked | 23 (2.1%) | 9 (7.0%) |
|  |  | Don't Remember | 44 (4.0%) | 1 (0.8%) |
| Provide advice on benefits of quitting | HCP | All the time | 582 (54.5%) | 175 (74.2%) |
|  |  | Most of the time | 305 (28.6%) | 45 (19.1%) |
|  |  | Half of the time | 61 (5.7%) | 6 (2.5%) |
|  |  | Not much of the time | 93 (8.7%) | 10 (4.2%) |
|  |  | Never | 26 (2.4%) | 0 (0.0%) |
| Offer personalised advice on how to stop smoking | HCP | All the time | 294 (27.9%) | 93 (39.4%) |
|  |  | Most of the time | 302 (28.7%) | 76 (32.2%) |
|  |  | Half of the time | 139 (13.2%) | 26 (11.0%) |
|  |  | Not much of the time | 209 (19.9%) | 33 (14.0%) |
|  |  | Never | 108 (10.3%) | 8 (3.4%) |
| Refer to Quitline or other stop smoking service | HCP | All the time | 388 (36.6%) | 115 (49.4%) |
|  |  | Most of the time | 321 (30.3%) | 69 (29.6%) |
|  |  | Half of the time | 137 (12.9%) | 18 (7.7%) |
|  |  | Not much of the time | 146 (13.8%) | 29 (12.4%) |
|  |  | Never | 68 (6.4%) | 2 (0.9%) |
| Record passive smoking status at first antenatal visit | HCP | All the time | 447 (46.5%) | 125 (55.8%) |
|  |  | Most of the time | 137 (14.2%) | 43 (19.2%) |
|  |  | Half of the time | 49 (5.1%) | 8 (3.6%) |
|  |  | Not much of the time | 138 (14.3%) | 21 (9.4%) |
|  |  | Never | 191 (19.9%) | 27 (12.1%) |
| Asked at booking whether regularly exposed to passive smoke | Women | Yes |  | 92 (71.3%) |
|  |  | No |  | 23 (17.8%) |
|  |  | Don't remember |  | 14 (10.9%) |
| Refer partner to Quitline/other if they smoke | HCP | All the time | 160 (15.3%) | 55 (23.7%) |
|  |  | Most of the time | 213 (20.4%) | 60 (25.9%) |
|  |  | Half of the time | 130 (12.5%) | 26 (11.2%) |
|  |  | Not much of the time | 255 (24.4%) | 64 (27.6%) |
|  |  | Never | 286 (27.4%) | 27 (11.6%) |
| Ask women if they attended Quitline/other appointment | HCP | All the time | 139 (14.0%) | 51 (22.6%) |
|  |  | Most of the time | 198 (19.9%) | 84 (37.2%) |
|  |  | Half of the time | 147 (14.8%) | 16 (7.1%) |
|  |  | Not much of the time | 220 (22.2%) | 43 (19.0%) |
|  |  | Never | 289 (29.1%) | 32 (14.2%) |
| Use 'Ask, Advise and Help' brief advice model at every visit | HCP | All the time | 152 (15.6%) | 47 (20.7%) |
|  |  | Most of the time | 217 (22.2%) | 87 (38.3%) |
|  |  | Half of the time | 136 (13.9%) | 24 (10.6%) |
|  |  | Not much of the time | 160(16.4%) | 34 (15.0%) |
|  |  | Never | 311 (31.9%) | 35 (15.4%) |
| Offer all women exhaled breath CO reading | HCP | All the time | 38 (4.5%) | 14 (8.1%) |
|  |  | Most of the time | 38 (4.5%) | 25 (14.5%) |
|  |  | Half of the time | 37 (4.4%) | 10 (5.8%) |
|  |  | Not much of the time | 64 (7.6%) | 25 (14.5%) |
|  |  | Never | 667 (79.0%) | 99 (57.2%) |
| CO Breath test offered | Women | Yes | 26 (2.4%) | 3 (2.3%) |
|  |  | No | 1022 (93.2%) | 102 (79.1%) |
|  |  | Don't Remember | 48 (4.4%) | 4 (3.1%) |
| Quit smoking brochure received and read | Women | Yes I read the brochure |  | 58 (45.0%) |
|  |  | Yes, but I did not read the brochure |  | 26 (20.1%) |
|  |  | No, but I was given a different brochure on this topic |  | 2 (1.6%) |
|  |  | No, I was not given a brochure on this topic |  | 30 (23.3%) |
|  |  | Don't remember |  | 13 (10.1%) |
| Quit smoking brochure provided | HCP | Yes |  | 173 (72.1%) |
|  |  | No, I provide a different brochure on smoking cessation |  | 13 (5.4%) |
|  |  | No, I do not provide any brochure on smoking cessation |  | 17 (7.1%) |
|  |  | I don't know about this brochure |  | 37 (15.4%) |
| Assess for risk factors for FGR early in pregnancy | HCP | All the time | 597 (59.2%) | 194 (84.0%) |
|  |  | Most of the time | 257 (25.5%) | 27 (11.7%) |
|  |  | Half of the time | 40 (4.0%) | 3 (1.3%) |
|  |  | Not much of the time | 76 (7.5%) | 7 (3.0%) |
|  |  | Never | 38 (3.8%) | 0 (0.0%) |
| Assess for risk factors for FGR at visits from 24 weeks’ | HCP | All the time | 630 (61.2%) | 169 (72.5%) |
|  |  | Most of the time | 263 (25.5%) | 44 (18.9%) |
|  |  | Half of the time | 53 (5.1%) | 10 (4.3%) |
|  |  | Not much of the time | 57 (5.5%) | 8 (3.4%) |
|  |  | Never | 27 (2.6%) | 2 (0.9%) |
| SFH measure at visits from 24 weeks’ | HCP | All the time | 875 (85.0%) | 212 (90.6%) |
|  |  | Most of the time | 91 (8.8%) | 16 (6.8%) |
|  |  | Half of the time | 16 (1.6%) | 2 (0.9%) |
|  |  | Not much of the time | 21 (2.0%) | 4 (1.7%) |
|  |  | Never | 27 (2.6%) | 0 (0.0%) |
| SFH measured (at all antenatal appointments from 28 weeks’) | Woman | Yes, at every antenatal appointment (from 28 weeks' gestation) | 939 (85.7%) | 105 (81.4%) |
|  |  | Yes, but only at one or some antenatal appointments (from 28 week's gestation) | 123 (11.2%) | 22 (17.1%) |
|  |  | No | 29 (2.6%) | 2 (1.6%) |
|  |  | Don’t remember | 5 (0.5%) | 0 (0.0%) |
| Plot SFH on growth chart | HCP | All the time | 228 (22.8%) | 114 (49.4%) |
|  |  | Most of the time | 86 (8.6%) | 51 (22.1%) |
|  |  | Half of the time | 57 (5.7%) | 22 (9.5%) |
|  |  | Not much of the time | 137 (13.7%) | 25 (10.8%) |
|  |  | Never | 492 (49.2%) | 19 (8.2%) |
| Refer for growth scans if at increased risk | HCP | All the time | 481 (51.3%) | 139 (69.2%) |
|  |  | Most of the time | 204 (21.8%) | 34 (16.9%) |
|  |  | Half of the time | 57 (6.1%) | 5 (2.5%) |
|  |  | Not much of the time | 79 (8.4%) | 13 (6.5%) |
|  |  | Never | 116 (12.4%) | 10 (5.0%) |
| Growth Matters brochure received and read | Women | Yes I read the brochure |  | 63 (48.8%) |
|  |  | Yes, but I did not read the brochure |  | 13 (10.1%) |
|  |  | No, but I was given a different brochure on this topic |  | 4 (3.1%) |
|  |  | No, I was not given a brochure on this topic |  | 34 (26.4%) |
|  |  | Don't remember |  | 15 (11.6%) |
| Growth Matters brochure provided | HCP | Yes |  | 126 (53.4%) |
|  |  | No, I provide a different brochure on FGR cessation |  | 5 (2.1%) |
|  |  | No, I do not provide any brochure on FGR |  | 34 (14.4%) |
|  |  | I don't know about this brochure |  | 71 (30.1%) |
| Discuss importance of reporting DFM, each visit from 28 weeks’ | HCP | All the time | 884 (83.3%) | 233 (93.2%) |
|  |  | Most of the time | 138 (13.0%) | 13 (5.2%) |
|  |  | Half of the time | 17 (1.6%) | 2 (0.8%) |
|  |  | Not much of the time | 17 (1.6%) | 2 (0.8%) |
|  |  | Never | 5 (0.5%) | 0 (0.0%) |
| Baby's movements discussed, each visit from 28 weeks’ | Women | Yes, at every antenatal appointment | 752 (69%) | 107 (82.9%) |
|  |  | Yes, but only at one or some antenatal appointments | 268 (24%) | 13 (10.1%) |
|  |  | No | 49 (4%) | 7 (5.4%) |
|  |  | Don’t remember | 27 (2%) | 2 (1.6%) |
| From 28 weeks’, how often CTG within 2 hours if concern about DFM | HCP | All the time | 869 (79.9%) | 201 (81.7%) |
|  |  | Most of the time | 198 (18.2%) | 41 (16.7%) |
|  |  | Half of the time | 12 (1.1%) | 0 (0.0%) |
|  |  | Not much of the time | 6 (0.6%) | 4 (1.6%) |
|  |  | Never | 2 (0.2%) | 0 (0.0%) |
| Movements Matter brochure received and read | Women | Yes I read the brochure | 454 (41.4%) | 95 (73.6%) |
|  |  | Yes, but I did not read the brochure | 54 (4.9%) | 7 (5.4%) |
|  |  | No, but I was given a different brochure on this topic | 91 (8.3%) | 6 (4.7%) |
|  |  | No, I was not given a brochure on this topic | 277 (25.3%) | 12 (9.3%) |
|  |  | Don't remember | 220 (20.1%) | 9 (7.0%) |
| Movements Matter brochure provided | HCP | Yes | 496 (43.2%) | 211 (85.4%) |
|  |  | No, I provide a different brochure on DFM | 170 (14.8%) | 1 (0.4%) |
|  |  | No, I do not provide any brochure on DFM | 232 (20.2%) | 13 (5.3%) |
|  |  | I don't know about this brochure | 250 (21.8%) | 22 (8.9%) |
| Provide information and discuss safe sleep position by 28 weeks’ | HCP | All the time | 216 (20.4%) | 197 (79.4%) |
|  |  | Most of the time | 214 (20.2%) | 24 (9.7%) |
|  |  | Half of the time | 95 (9.0%) | 6 (2.4%) |
|  |  | Not much of the time | 223 (21.1%) | 11 (4.4%) |
|  |  | Never | 311 (29.4%) | 10 (4.0%) |
| Discuss safe going-to-sleep position at every visit from 28 weeks’ | HCP | All the time | 241 (22.8%) | 168 (66.4%) |
|  |  | Most of the time | 267 (25.2%) | 45 (17.8%) |
|  |  | Half of the time | 151 (14.3%) | 18 (7.1%) |
|  |  | Not much of the time | 229 (21.6%) | 17 (6.7%) |
|  |  | Never | 170 (16.1%) | 5 (2.0%) |
| Importance of sleeping on side in late pregnancy discussed (at all antenatal appointments from 28 weeks’) | Women | Yes, at every antenatal appointment (after 28 weeks) | 285 (26.0%) | 71 (55.0%) |
|  |  | Yes, but only at one or some antenatal appointments (after 28 weeks) | 398 (36.3%) | 49 (38.0%) |
|  |  | No | 284 (25.9%) | 5 (3.9%) |
|  |  | Don't remember | 129 (11.8%) | 4 (3.1%) |
| Sleep-on-side brochure received and read | Women | Yes I read the brochure |  | 104 (80.6%) |
|  |  | Yes, but I did not read the brochure |  | 10 (7.8%) |
|  |  | No, but I was given a different brochure on this topic |  | 1 (0.8%) |
|  |  | No, I was not given a brochure on this topic |  | 7 (5.4%) |
|  |  | Don't remember |  | 7 (5.4%) |
| Sleep-on-side brochure provided | HCP | Yes |  | 205 (81.3%) |
|  |  | No, I provide a different brochure on side-sleeping |  | 5 (2.0%) |
|  |  | No, I do not provide any brochure on side-sleeping |  | 16 (6.3%) |
|  |  | I don't know about this brochure |  | 26 (10.3%) |
| Assess for stillbirth risk factors first antenatal visit | HCP | All the time | 436 (44.5%) | 169 (72.5%) |
|  |  | Most of the time | 213 (21.7%) | 46 (19.7%) |
|  |  | Half of the time | 66 (6.7%) | 4 (1.7%) |
|  |  | Not much of the time | 138 (14.1%) | 13 (5.6%) |
|  |  | Never | 127 (13.0%) | 1 (0.4%) |
| Reassess for stillbirth risk factors 34 to 36+6 weeks gestation | HCP | All the time | 265 (26.3%) | 112 (47.1%) |
|  |  | Most of the time | 210 (20.9%) | 63 (26.5%) |
|  |  | Half of the time | 97 (9.6%) | 24 (10.1%) |
|  |  | Not much of the time | 205 (20.4%) | 29 (12.2%) |
|  |  | Never | 230 (22.8%) | 10 (4.2%) |
| Discuss birth planning according to risk status | HCP | All the time | 296 (29.1%) | 110 (45.8%) |
|  |  | Most of the time | 250 (24.6%) | 71 (29.6%) |
|  |  | Half of the time | 103 (10.1%) | 22 (9.2%) |
|  |  | Not much of the time | 186 (18.3%) | 31 (12.9%) |
|  |  | Never | 181 (17.8%) | 6 (2.5%) |
| Possibility of having a planned birth discussed | Women | Yes, at antenatal appointments late in my pregnancy (around 34-36 weeks) | 310 (28%) | 45 (34.9%) |
|  |  | Yes, at antenatal appointments early in my pregnancy | 143 (13%) | 18 (14.0%) |
|  |  | Yes, at antenatal appointments both early and late in my pregnancy | 92 (8%) | 18 (14.0%) |
|  |  | No | 492 (45%) | 34 (26.4%) |
|  |  | Don't remember | 59 (5%) | 14 (10.9%) |
| Provide individual information about birth timing based on stillbirth risk | HCP | All the time | 303 (29.8%) | 141 (57.6%) |
|  |  | Most of the time | 256 (25.2%) | 70 (28.6%) |
|  |  | Half of the time | 112 (11.0%) | 11 (4.5%) |
|  |  | Not much of the time | 167 (16.4%) | 15 (6.1%) |
|  |  | Never | 179 (17.6%) | 8 (3.3%) |
| Involved as much as you wanted to be when making decisions and choosing options about the timing of your baby’s birth | Women | Yes | 825 (75.3%) | 97 (75.2%) |
|  |  | No | 218 (19.9%) | 23 (17.8%) |
|  |  | Don't remember | 53 (4.8%) | 9 (7.0%) |
