# Supplementary material for: Experiences of antenatal care practices to reduce stillbirth: surveys of women and healthcare professionals pre-post implementation of the Safer Baby Bundle
Source: BMC Pregnancy Childbirth. 2024 Aug 1;24:520. doi: 10.1186/s12884-024-06712-8 (PMC11295589; doi:10.1186/s12884-024-06712-8)
Supplement: Supplementary file 4 — Supplementary Material 4 [file 12884_2024_6712_MOESM4_ESM.docx]

**Additional file 4.**

Healthcare professionals’ change in knowledge and confidence for having conversations with women about the five SBB elements.

|  |  | **Agree n (%)** | |  |
| --- | --- | --- | --- | --- |
| **Statement** | **Element** | **Pre-SBB** | **Post-SBB** | **p-value** |
| I have adequate time to provide appropriate care | 1 | 382 (36.4%) | 135 (53.4%) | <0.001 |
|  | 2 | 505 (48.0%) | 165 (64.7%) | <0.001 |
|  | 3 | 823 (73.3%) | 213 (81.3%) | 0.007 |
|  | 4 | 626 (58.1%) | 209 (80.1%) | <0.001 |
|  | 5 | 561 (53.3%) | 173 (68.9%) | <0.001 |
| I feel confident about my level of knowledge | 1 | 564 (52.5%) | 228 (87.0%) | <0.001 |
|  | 2 | 633 (59.4%) | 238 (90.5%) | <0.001 |
|  | 3 | 1,022 (91.0%) | 260 (97.7%) | <0.001 |
|  | 4 | 691 (63.4%) | 256 (96.2%) | <0.001 |
|  | 5 | 531 (49.5%) | 222 (85.4%) | <0.001 |
| I feel comfortable discussing this with women | 1 | 804 (74.9%) | 241 (92.0%) | <0.001 |
|  | 2 | 832 (78.1%) | 253 (95.8%) | <0.001 |
|  | 3 | 1,064 (94.7%) | 263 (98.9%) | 0.003 |
|  | 4 | 855 (78.8%) | 260 (98.1%) | <0.001 |
|  | 5 | 640 (59.9%) | 210 (81.1%) | <0.001 |
| I have concerns that this conversation will cause anxiety for women | 1 | 466 (43.5%) | 107 (40.8%) | 0.44 |
|  | 2 | 402 (37.7%) | 84 (32.1%) | 0.091 |
|  | 3 | 304 (27.0%) | 68 (25.6%) | 0.63 |
|  | 4 | 229 (21.0%) | 36 (13.5%) | 0.006 |
|  | 5 | 568 (52.9%) | 147 (56.5%) | 0.30 |
| I have concerns that this conversation will negatively impact on my relationship with the woman | 1 | 270 (25.2%) | 48 (18.3%) | 0.020 |
|  | 2 | 83 (7.8%) | 16 (6.1%) | 0.36 |
|  | 3 | 70 (6.2%) | 12 (4.5%) | 0.29 |
|  | 4 | 56 (5.1%) | 8 (3.0%) | 0.14 |
|  | 5 | 129 (12.0%) | 25 (9.6%) | 0.28 |

Agree (agree/strongly agree). There were no missing values and response for ‘not applicable’ have been excluded so they do not contribute to the numerator or denominator.
