# Supplementary material for: Experiences of antenatal care practices to reduce stillbirth: surveys of women and healthcare professionals pre-post implementation of the Safer Baby Bundle
Source: BMC Pregnancy Childbirth. 2024 Aug 1;24:520. doi: 10.1186/s12884-024-06712-8 (PMC11295589; doi:10.1186/s12884-024-06712-8)
Supplement: Supplementary file 5 — Supplementary Material 5 [file 12884_2024_6712_MOESM5_ESM.docx]

**Additional file 5.**

Post-SBB healthcare professionals’ level of agreement with statements and impressions considering the SBB initiative.

| **Item** | **Level of agreement/impression** | **Post-SBB n (%)** |
| --- | --- | --- |
| The SBB has improved the quality of antenatal care I provide | Disagree | 29 (10.9%) |
|  | Neutral | 52 (19.5%) |
|  | Agree | 186 (69.7%) |
| The SBB has improved the quality of antenatal care provided by the service in which I work | Disagree | 24 (9.0%) |
|  | Neutral | 55 (20.6%) |
|  | Agree | 188 (70.4%) |
| The SBB is effective | Disagree | 18 (6.7%) |
|  | Neutral | 67 (25.1%) |
|  | Agree | 182 (68.2%) |
| The SBB recommendations are evidence-based. | Disagree | 11 (4.1%) |
|  | Neutral | 39 (14.7%) |
|  | Agree | 216 (81.2%) |
| I regularly use the SBB to inform my practice. | Disagree | 15 (5.6%) |
|  | Neutral | 41 (15.4%) |
|  | Agree | 210 (78.9%) |
| I have enough time in my everyday practice to follow the SBB recommendations | Disagree | 51 (19.2%) |
|  | Neutral | 56 (21.1%) |
|  | Agree | 159 (59.8%) |
| I feel like the recommendations of the SBB have become part of my routine practice. | Disagree | 11 (4.1%) |
|  | Neutral | 29 (10.9%) |
|  | Agree | 226 (85.0%) |
| The SBB elements of care have been well implemented at my maternity service. | Disagree | 14 (5.3%) |
|  | Neutral | 53 (19.9%) |
|  | Agree | 199 (74.8%) |
| Impression of the impact of implementing the SBB elements at your service: Overall | Negative | 16 (6.0%) |
|  | Neutral | 49 (18.4%) |
|  | Positive | 202 (75.7%) |
| Impression of the impact of implementing: Smoking Cessation (Element 1) | Negative | 9 (3.4%) |
|  | Neutral | 86 (32.2%) |
|  | Positive | 172 (64.4%) |
| Impression of the impact of implementing: FGR (Element 2) | Negative | 14 (5.2%) |
|  | Neutral | 53 (19.9%) |
|  | Positive | 200 (74.9%) |
| Impression of the impact of implementing: DFM (Element 3) | Negative | 16 (6.0%) |
|  | Neutral | 35 (13.1%) |
|  | Positive | 216 (80.9%) |
| Impression of the impact of implementing: Sleep-on-side (Element 4) | Negative | 2 (0.7%) |
|  | Neutral | 40 (15.0%) |
|  | Positive | 225 (84.3%) |
| Impression of the impact of implementing: Timing of birth (Element 5) | Negative | 40 (15.0%) |
|  | Neutral | 82 (30.7%) |
|  | Positive | 145 (54.3%) |

Level of agreement- Disagree (strongly disagree/disagree), Neutral, Agree (strongly agree/agree); and impression- Negative (very negative/negative), Neutral, Positive (positive/very positive).
